# The Impact of Exercise Training Plus Dietary Interventions on Ectopic Fat in Population with Overweight/Obesity with and without Chronic Disease: A Systematic Review, Meta-analysis, and Metaregression of Randomized Clinical Trials

**DOI:** 10.1016/j.cdnut.2025.104574

**Published:** 2025-02-21

**Authors:** Fatemeh Kazeminasab, Mohammad Hossein Mahboobi, Motahareh Mohebinejad, Maedeh Nojoumi, Saba Belyani, Donny M Camera, Sajjad Moradi, Reza Bagheri

**Affiliations:** 1 Department of Physical Education and Sports Science, Faculty of Humanities, University of Kashan, Kashan, Iran; 2 Department of Nutrition, Faculty of Medicine, Mashhad University of Medical Sciences, Mashhad, Iran; 3 Human Nutrition Program, Department of Human Sciences, The Ohio State University, Columbus, OH, United States; 4 Department of Health and Biostatistics, Swinburne University, Melbourne, Australia; 5 Human Nutrition Program, Department of Human Sciences, The Ohio State University, Columbus, USA; 6 Department of Nutrition and Food Sciences, Maragheh University of Medical Sciences, Maragheh, Iran; 7 Department of Exercise Physiology, University of Isfahan, Isfahan, Iran

**Keywords:** dietary intervention, exercise training, ectopic fat, obesity, liver fat

## Abstract

**Background:**

The growing prevalence of obesity and related chronic diseases has led to increased interest in interventions targeting ectopic fat reduction to which its accumulation is linked to metabolic dysfunction.

**Objectives:**

This study aimed to evaluate the effects of combined exercise training combined with dietary interventions compared with dietary interventions alone on ectopic fat [visceral fat area (VFA), liver fat, intramuscular fat (IMF), pancreatic fat, renal sinus fat, and pericardial and epicardial fats] in adults with overweight and obesity, both with and without chronic diseases.

**Methods:**

Web of Science, Scopus, and PubMed were searched for original articles up to 1 March, 2024, that included exercise compared with control interventions on body weight and ectopic fat in adults with overweight or obesity. Weighted mean differences (WMD) for body weight, liver fat, pancreatic fat, and renal sinus fat and standardized mean differences (SMD) for VFA, IMF, pericardial and epicardial fats, and 95% confidence intervals were determined using random-effects models.

**Results:**

Thirty-two studies, including 1488 participants and 38 intervention groups, met the inclusion criteria. The combined intervention of exercise and diet did not reduce body weight (WMD = –0.23 kg, *P* = 0.180), liver fat (WMD = 0.05%, *P* = 0.730), IMF (SMD = –0.08, *P* = 0.640), pericardial and epicardial fats (SMD = –0.12, *P* = 0.280), pancreatic fat (WMD = –0.24%, *P* = 0.370), and renal sinus fat (WMD = 0.01 cm^2^, *P* = 0.170) when compared with a diet-only group. Interestingly, exercise combined with diet significantly reduced VFA in participants with obesity (SMD = –0.12, *P* = 0.040) and healthy males (SMD = –0.33, *P* = 0.001) when compared with a diet-only group.

**Conclusions:**

The findings suggest that combined exercise and dietary interventions did not lead to significant reductions in most ectopic fat depots when compared with diet alone. However, a modest reduction in VFA was observed in participants with obesity and healthy males. These results highlight the nuanced impact of exercise in combination with dietary interventions and the need to consider specific fat depots and participant characteristics in obesity management strategies.

The trial was registered at PROSPERO as CRD42024546770.

## Introduction

In individuals with obesity, lipid deposition can occur in nonadipose tissues such as the muscle, liver, and pancreas when adipose tissue’s capacity to buffer and store excess fat is impaired [1]. This phenomenon, known as ectopic fat deposition, is associated with insulin resistance and metabolic risk. The significance of ectopic fat aligns with findings in individuals afflicted with lipodystrophy, who exhibit pronounced insulin resistance. Among this population, the absence of adipose tissue results in the accumulation of lipids in extra adipose sites [[2], [3], [4]]. The crucial aspect to consider is that body fat distribution is frequently more important than total body fat because variations in the distribution of adipose tissue across different regions may play a significant role in the variability observed in metabolic risk profiles among individuals who possess similar BMIs [5]. Although abdominal obesity has been extensively studied, emerging evidence suggests that the accumulation of excess fat around the heart and coronary arteries could have particularly adverse effects on cardiovascular health [6]. This condition is linked to reduced cardiorespiratory fitness and an increased likelihood of developing coronary artery disease [[7], [8], [9], [10], [11], [12], [13]]. Furthermore, there is a correlation between heightened visceral fat and the development of adverse cardiometabolic conditions, including the accumulation of fat in the liver, pericardium, pancreas, kidneys, and skeletal muscle [[14], [15], [16]]. In individuals with obesity, visceral adipose tissue (VAT) and total abdominal fat can be significantly reduced with a moderate 5% initial body mass reduction [17].

To facilitate the loss of body mass, guidelines for treating obesity advocate a holistic approach to lifestyle modification, incorporating a calorie-restricted (CR) diet and heightened levels of physical activity [18]. However, this integrated approach may only sometimes be advised. However, diet is regarded as the fundamental component in obesity management, and the portrayal of exercise/physical activity as less effective in certain media narratives has contributed to misconceptions about its benefits [19,20]. Consequently, the importance of physical activity is often underestimated [21]. Previous research suggests that exercise training induces a caloric deficit and positively affects mitochondrial function within skeletal muscle [22]. Studies show that exercise can increase mitochondrial size, as observed in trained athletes [23,24] and healthy young adults [25], whereas reduced energy metabolism is associated with smaller mitochondrial size [26]. Exercise and diet-based lifestyle treatments can successfully reduce ectopic fat and diminish triglyceride deposition in nonadipose tissues, such as the liver, heart, pancreas, and intracellular lipids [27]. However, there is limited understanding of how combined dietary and exercise interventions compare with dietary approaches alone regarding long-term fat reduction across various ectopic fat depots, including the renal sinus, pericardium, epicardium, liver, intramuscular, and pancreas [[28], [29], [30], [31]].

Previous studies have predominantly focused on individual fat depots or have lacked a direct comparison between combined exercise and dietary interventions compared with diet alone [32]. This study aims to fill this gap by comprehensively assessing the effects of combined exercise and dietary interventions compared with diet alone on various ectopic fat depots, including visceral fat, intramuscular fat (IMF), intrahepatic fat (IHL), pericardial and epicardial fats, pancreatic fat, and renal sinus fat, in populations with overweight and obesity. By employing meta-analytic methods, this study offers a more robust understanding of the intervention effects, thereby contributing to a more personalized and effective approach to obesity management.

## Methods

### Trial registration

The present systematic review and meta-analysis was conducted based on the guidelines set by the PRISMA guidelines [33] and followed the additional guidance provided by the Cochrane Handbook of Systematic Reviews of Interventions [34].

### Inclusion and exclusion criteria

The following inclusion criteria were applied: *1*) English language articles; *2*) studies of human participants with overweight/obesity; *3*) studies where the experimental group underwent a combination of exercise training and dietary intervention and was compared with diet only as the control group; *4*) randomized control trials; *5*) studies with assessments of liver fat%, visceral fat area (VFA), IMF, pericardial and epicardial fats, pancreatic fat, and renal sinus fat with pre- and postintervention or change scores reported.

Exclusion criteria included: *1*) studies written in a non-English language; *2*) nonoriginal and nonexperimental research such as case-control studies, cross-sectional studies, study protocols, conference proceedings, letters to the editor, reviews, and meta-analyses; *3*) studies where the dietary interventions of the exercise group compared with exercise only as a control group; *4*) animal studies; and *5*) nonrandomized studies were excluded from the review.

### Search strategy and locate studies

To achieve comprehensive coverage, a comprehensive search was conducted across electronic databases, including Scopus, PubMed, and Web of Science. Two reviewers independently identified published research articles through 1 March, 2024.

Two reviewers independently identified published research articles using the following key words: (“type 2 diabetes” or “non-insulin-dependent diabetes∗” or “type II diabetes∗” or “diabetes mellitus, type 2” or “diabetes mellitus” or “overweight” or “obese” or “obesity” or “metabolic syndrome” or “HOMA-IR” or “insulin resistance” or “homeostatic model assessment for insulin resistance” AND “weight loss” or “caloric restriction” or “diet” or “low calorie diet” or “dietary” or “calorie restricted diet” or “energy restricted diet” or “very low-calorie diet” AND “physical activity” or “exercise training” or “exercise” or “training” or “athletes” or “aerobic training” or “aerobic exercise” or “endurance training” or “endurance exercise” or “resistance training” or “resistance exercise” or “strength training” or “strength exercise” or “combined training” or “combined exercise” or “concurrent training” or “concurrent exercise” or “exercise therapy” or “sports” or “lifestyle intervention” or “anaerobic training”). The following keywords (“hepatic lipid” or “fatty liver” or “hepatic fat” or “intra hepatic lipid” or “intra hepatic fat” or “IHTG” or “IHL” or “intra hepatic triglyceride” or “hepatic lipid content” or “hepatic fat content” or “hepatic lipid fraction” or “liver lipid content” or “liver fat content” or “hepatic fat fraction” or “hepatic lipid accumulation” or “hepatic fat accumulation”) were used for liver fat, keywords (“muscular fat” or “muscle fat” or “muscle lipid” or “intramyocellular lipid” or “muscular lipid” or “skeletal muscle fat” or “skeletal muscle lipid” or “intramyocellular fat” or “muscular triglycerides” or “muscle fat fraction” or “muscle lipid fraction” or “intramyocellular triglycerides” or “muscle fat content” or “muscle lipid content” or “IMTG” or “IMCL”) for IMF, and keywords (“visceral adipose tissue” or “VAT” or “abdominal adipose tissue” or “abdominal fat” or “visceral fat” or “ectopic adipose tissue” or “ectopic fat”) for visceral fat. The filters, including English, human, and journal, were applied. In addition, a manual search of the reference lists of all included studies was accomplished on Google Scholar. To ensure comprehensive coverage of relevant records, the reference lists of all included studies were examined for any additional sources that may have been missed in the initial electronic search. To ensure that all eligible studies were included in the present meta-analysis study, the searches were conducted independently by 2 authors, and any disagreements were determined by discussion with another researcher.

### Study selection

Studies were included if the exercise intervention duration was ≥2 wk. Trials involving supervised aerobic, resistance, or combined training were included. Studies with CR diets and diets with the aim of losing body weight were included in the present meta-analysis. Studies using noninvasive imaging techniques such as computed tomography (CT), MRI, and hydrogen-based magnetic resonance spectroscopy for ectopic fat were included in this meta-analysis [[35], [36], [37], [38]]. The present systematic review and meta-analysis study focused on interventions based on intervention (combination of diet and exercise compared with diet only). For the current study, exercise was defined as any coordinated or supervised exercise program aiming to reduce body mass or body fat.

**FIGURE 1:**
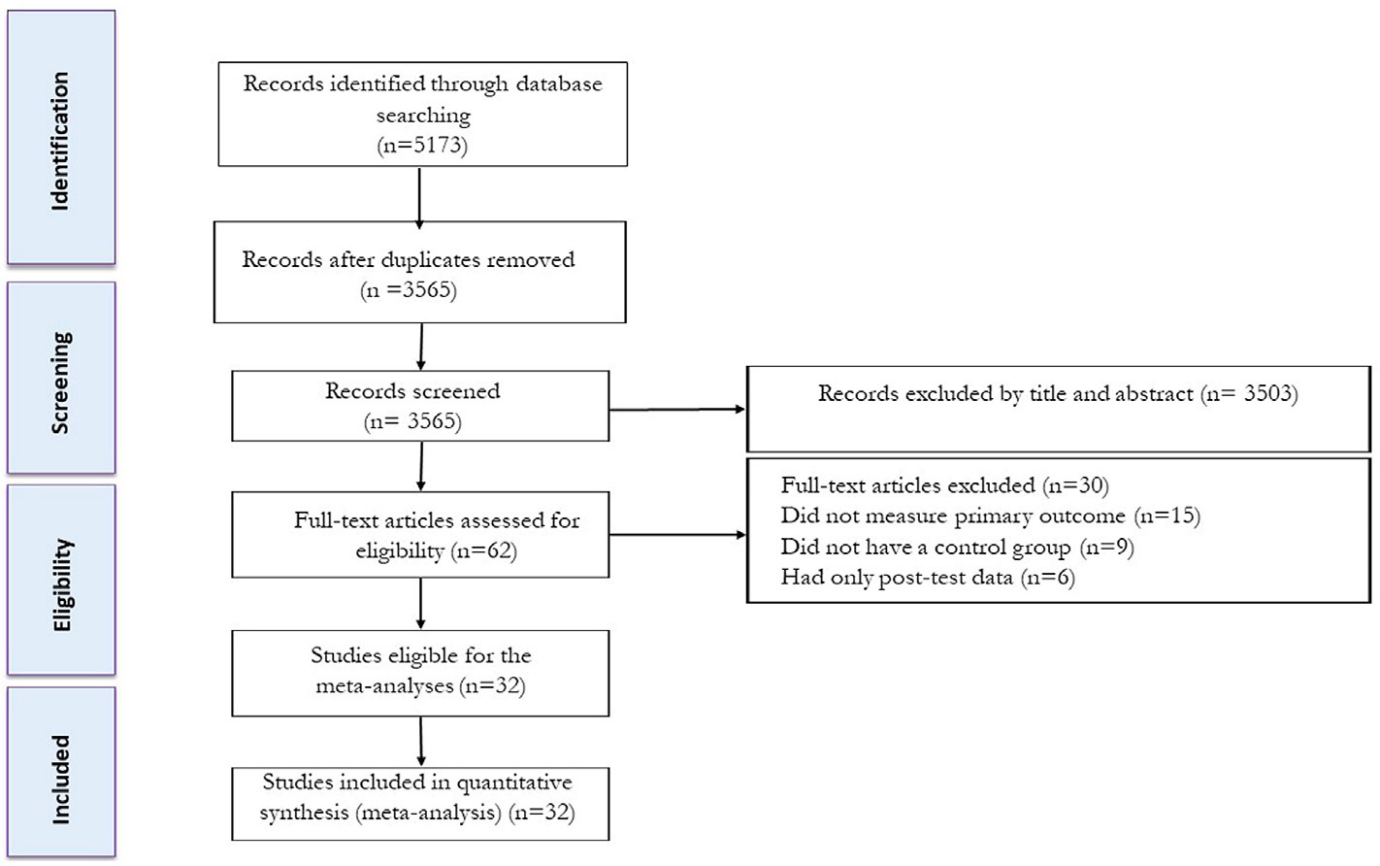
Flow diagram of systematic literature search.

The study selection process is shown in Figure 1. Articles were independently evaluated after the removal of duplicate studies, titles, and abstracts, in which full texts were reviewed by 2 reviewers to determine eligibility. Any disagreements were resolved through discussion with another author. The following study characteristics were extracted: *1*) participant characteristics, including health condition, biological sex, age, BMI, and sample size; *2*) diet characteristics; and *3*) exercise characteristics, and duration of intervention (wk). Participants with overweight (BMI ≥ 25 kg/m^2^) and obesity (BMI ≥ 30) were included in the present study.

### Quality assessment

The risk of bias was evaluated using the Physiotherapy Evidence Database (PEDro) scale [39]. We excluded 2 items (lack of blinding of participants and intervention providers) from the original 11-item scale because participants and intervention providers could not be blinded to the assigned diet conditions during studies. The scale used for the current study consisted of 9 items: *1*) specified eligibility criteria, *2*) randomly assigned participant allocation, *3*) concealed allocation, *4*) similarity of groups at baseline, *5*) blinding of all assessors, *6*) evaluated outcomes in 85% of participants, *7*) intention-to-treat analysis, *8*) reporting of statistical comparisons between groups, *9*) and point measures and measures of variability (Supplemental Table 1).

### Data extraction

The data were extracted by 2 authors. For each outcome (body weight, liver fat, VFA, IMF, pericardial and epicardial fats, pancreatic fat, and renal sinus fat), pre- and postintervention (means and SDs), or mean differences and associated SDs were entered into the meta-analyses to generate forest plots. If the means and SDs were not reported, the SDs were calculated from SEMs and medians and IQRs [[40], [41], [42]].

### Statistical analysis

Meta-analyses were performed using the comprehensive meta-analysis software (version 2.0, Biostat Inc.) to calculate weighted mean differences (WMDs) or standardized mean differences (SMDs) and 95% confidence intervals (CIs) for outcomes using random-effects models. Results were pooled using random-effects models, based on the assumption that heterogeneity was likely from a clinical perspective and may have affected the findings [43]. The units of measurement of body weight (kg), liver fat (%), pancreatic fat (%), and renal sinus fat (cm^2^) were the same. Therefore, for these outcomes, WMD was used. The units of measurement of VFA (cm^2^, cm^3^, or g), IMF (cm^2^ or g), and pericardial and epicardial fat (cm^2^ or cm^3^) were different across the studies included in the present study; therefore, SMD was used for these outcomes. Effect sizes were calculated to compare the combined exercise training and dietary interventions compared with the independent effect of diet on body weight and ectopic fat (liver fat, VFA, IMF, pericardial and epicardial fats, pancreatic fat, and renal sinus fat) in individuals with overweight and obesity.

Heterogeneity was evaluated by using the *I*^2^ statistic; significance was set at *P* < 0.05. According to Cochrane guidelines, *I*^2^ statistics were interpreted as follows: <25% as very low, 25%–50% as low, 50%–75% as moderate, and >75% as high heterogeneity [44].

Subgroup analyses were performed as follows: *1*) health status (adults with or without metabolic diseases), *2*) type of exercise (aerobic, resistance, or combined exercises), *3*) intervention duration (short-term intervention ≤12 wk, or long-term interventions >12 wk), *4*) BMI [overweight (BMI ≥ 25), or obesity (BMI ≥ 30) adults], *5*) type of diet [caloric restriction, Paleolithic-type diet (PD), or Mediterranean diet], and *5*) gender (male or female).

Moreover, univariate metaregression analyses of body weight and ectopic fat (liver fat, VFA, IMF, pericardial and epicardial fats, pancreatic fat, and renal sinus fat) were conducted comparing diet alone as the control group.

### Sensitivity analysis

Sensitivity analyses were also conducted for all outcomes using the “remove 1” technique. This procedure assessed whether individual studies had a disproportionate impact on the results of the meta-analyses.

### Publication bias

Publication bias was detected through the visual interpretation of funnel plots. If publication bias was present, Egger’s tests were used as a confirmatory test. Significant publication bias was deemed apparent if *P* < 0.1 [45]. Sensitivity analyses were also conducted for all outcomes using the “remove 1” technique. This procedure assessed whether individual studies had a disproportionate impact on the results of the meta-analyses [46].

## Results

### Included studies

Our initial search strategy identified 1599 articles from Scopus, 2783 articles from Web of Science, and 1854 articles from PubMed. After eliminating duplicate records (1546) and screening titles and abstracts (initial screening), 62 studies were retrieved for a more detailed appraisal of the full texts (secondary screening). Thirty studies were excluded after reviewing full texts for the following reasons: *1*) 15 did not measure primary outcomes (liver fat, VFA, IMF, and heart fat); *2*) 9 did not have a diet group; *3*) 6 studies had only posttest data. A total of 32 studies, inclusive of 38 intervention groups, were included in the present systematic review and meta-analysis. A flow diagram of the systematic literature search is presented in Figure 1.

### Participant characteristics

A total of 1488 participants were included, with sample sizes ranging from 14 [47] to 278 [48]. The mean age of participants ranged from 25 [49] to 78 [50] y, and the mean BMIs of participants ranged from 25 to 44. The mean age of exercised and those who followed a diet was 51.39 ± 6.72 y, and the mean age of diet groups was 51.14 ± 7.32 y. The mean BMI of exercised and diet participants was 32.73 ± 4.63, and the mean BMI of diet groups was 32.39 ± 4.69. Both males and females were included in 19 studies [47,49,22,29,30,[51], [52], [53], [54], [55], [56], [57], [58], [59], [60], [61], [62], [63], [64]], females only in 9 studies [50,[65], [66], [67], [68], [69], [70], [71], [72]], and males only in 4 studies [48,[73], [74], [75]]. All participants were overweight/obese and with or without metabolic diseases. Table 1 presents the full details of participant characteristics.

**TABLE 1:** Characteristics of participants.

Study	Sample size (sex)	Health condition	Groups	Outcomes	Measure	Age (y), mean ± SD (range)	BMI (kg/m^2^) mean ± SD	Exercise intervention	Follow-up (wk)	Diet intervention
Brennan et al. (2022) [22]	41 (M&F)	Obese T2DM	Diet (CR) Com-Exe +Diet	IMF VFA	IMF: MRI VFA: MRI	Diet (CR): 70 ± 4.6 Com-Exe+Diet: 66.8 ± 3.4	Diet (CR): 36.1 ± 5.1 Com-Exe+Diet: 37.3 ± 5.4	Com-Exe: A-Exe 4–5 x/w (180 min/wk) R-Exe: week 8: 2 non-consecutive sessions/w for core muscles	26	Lose 10% of baseline body weight with reduction of 500–1000 kcal/d based on baseline body weight was prescribed in addition to a low-fat (<30% of kilocalories from fat) diet.
Brinkley et al. (2011) [65]	32 (F)	Obese Postmenopausal	Diet (CR) A-Exe_1_+Diet (CR) A-Exe_2_+Diet	Pericardial fat VFA	Pericardial fat: CT VFA: CT	Diet (CR): 57.6 ± 4.8 A-Exe_1_+Diet (CR): 57.3 ± 5.3 A-Exe_2_+Diet (CR): 59.4 ± 4.9	Diet (CR): 32.2 ± 4 A-Exe_1_+Diet (CR): 33.6 ± 4.5 A-Exe_2_+Diet (CR): 34.4 ± 4.7	A-Exe1 Moderate-intensity exercise, 3x/w, 15–55 min/session walking A-Exe2: Vigorous-intensity exercise, 3x/w, 90 min/w	12	Target energy intake: 800–1000 kcal/d
Cheng et al. (2017) [51]	45 (M&F)	NAFLD Prediabetes	Diet (CR) A-Exe+Diet	VFA LF	MRI	Diet (CR): 60 ± 4.1 A-Exe+ Diet: 60 ± 3.5	Diet (CR): 26.6 ± 2.7 A-Exe+Diet: 26.4 ± 2.9	A-Exe Progressive supervised A-Exe training (60%–75% VO_2max_ intensity), 2–3x/w with 30–60 min/sessions	48	CR with 37%–40% carbohydrate with 9–13 g as fiber, 35%–37% fat (SAFA 10%, MUFA 15%–20%, PUFA 10%) and 25%–27% protein. .
Chorell et al. (2021) [52]	26 (M&F)	Obese Postmenopausal T2DM	Diet (PD) Com-Exe+diet	LF IMF	LF: MRI IMF: MRI	Diet (PD): 58.3 ± 6.8 Com-Exe: 62.3 ± 4.5	Diet (PD): 31.6 ± 3 Com-Exe: 28.31.9 ± 3.7	Com-Exe: Aerobic and resistance exercises 3x/w	12	PD consumed ad libitum based on lean meat, fish, nuts, and vegetables. Dairy products, cereals, refined fats and sugars, and salt were excluded.
Cooper et al. (2012) [53]	90 (M&F)	Obese	Diet (CR) A-Exe+Diet	VFA	VFA: CT	Diet (CR): 47.5 ± 6.2 A-Exe+Diet: 46.8 ± 6.5	Diet (CR): 44 ± 6.6 A-Exe+Diet: 43.8 ± 4.8	A-Exe moderate-intensity physical activity for 5x/w, for 60 min/d duration	26	The target macronutrient composition was 20%–30% fat, 50%–55% carbohydrate, and 20%–25% protein to reduce energy intake to a target of 1200–2100 kcal/d.
Ezpeleta et al. (2023) [30]	39 (M&F)	NAFLD Obese	Diet (ADF) A-Exe+Diet	LF VFA	LF: MRI VFA: iDXA,	Diet (ADF): 44 ± 13.41 A-Exe+Diet (ADF): 44 ± 13.41	Diet (ADF): 36 ± 34.87 A-Exe+Diet (ADF): 37 ± 22.36	A-Exe 5 x/w, for 60 min at 65%–80% MHR	12	600 kcal as a dinner (between 17:00 and 20:00) on fast days and food ad libitum on alternating feast d.
Fayh et al. (2013) [54]	35 (M&F)	Obese	Diet (CR) A-Exe+Diet	VFA	VFA: CT	Diet (CR): 30.1 ± 5.5 A-Exe+Diet: 32.4 ± 7	Diet (CR): 34.7 ± 2.4 A-Exe+Diet: 34.7 ± 2.4	A-Exe 3 x/w for 30–60 min/session with 50%–70% HRR	4	Reducing energy consumption between 500 and 1000 kcal (2090 and 4180 kJ) per day
Ge et al. (2014) [50]	33 (F)	Overweight obese postmenopausal	Diet (CR) A-Exe+Diet	VFA	VFA: CT	Diet (CR): 50–78 A-Exe+Diet: 50–78	Diet (CR): ≥25 A-Exe+Diet: ≥25	A-Exe 3 x/w with treadmill	26	Restricted caloric intake by 500 kcal/d
Gepner et al. (2018) [48]	240 (M)	Dyslipidemia Obese	MD Com-Exe_1_+MD Com-Exe_2_+MD	LF Pancreatic fat Renal sinus fat IMAT Intrapericardial fat, Extra-pericardial fat VFA	MRI	MD: 48.4 ± 9.2 Com-Exe_1_+MD 48.4 ± 9.2 MD: 47.4 ± 9.3 Com-Exe_2_+MD 47.4 ± 9.3	MD: 30.8 ± 3.7 Com-Exe_1_+ MD: 30.8 ± 3.7 MD: 30.9 ± 4 Com-Exe_2_+ MD: 30.9 ± 4	Com-Exe: A-Exe 3 x/w 65%–80% MHR 20–45 min/session R-Exe: Total body for 3x/w, 1–2set(s)/session	78	MD Limit total fat to 30% of calories, with ≤10% saturated fat, max 300 mg cholesterol/d, increase fiber. Med/LCD: restricted carbs to <40 g/d, then raised to 70 g/d with more protein and fat in a Mediterranean diet. 28 g walnuts/d [160 kcal/84% fat, mainly PUFA (omega [ω] 3a-linolenic acid)] from month 3
Ghitea et al. (2021) [55]	75 (M&F)	Metabolic syndrome Obese	Diet (CR) A-Exe+Diet	VFA	VFA: CT	Diet (CR): 42.31 ± 17.95 A-Exe+Diet: 30.05 ± 9.40	Diet (CR): 30.95 ± 6.50 A-Exe+Diet: 31.42 ± 9.95	A-Exe A light exercise for cardiovascular stimulation, for 30–60 min, 2 to 3x/w	26	Clinical diet therapy for Metabolic syndrome intake of macronutrients in the percentage of 45%–55% carbohydrates, 25%–35% protein, and 15%–20% lipids, hypocaloric, with a reduction in caloric intake by 200 kcal
Goodpaster et al. (2010) [56]	100 (M&F)	Obese	Diet (CR) A-Exe+Diet	LF	LF: CT	Diet (CR): 47.5 ± 6.2 A-Exe+Diet: 46.1 ± 6.5	Diet (CR): 43.7 ± 5.9 A-Exe+Diet: 43.5 ± 4.8	A-Exe moderate intensity, 300 min/wk	26	Target energy intake = 1200–2100 kcal/d
Hays et al. (2006) [57]	22 (M&F)	Obese Impaired glucose tolerance	Diet (CR) A-Exe+Diet	VFA	VFA: CT	Diet (CR): 67.5 ± 7.29 A-Exe+Diet: 64.8 ± 6.63	Diet (CR): 31 ± 2.65 A-Exe+Diet0: 30.8 ± 3.64	A-Exe Exercise 4 d a week, for 45 min a day in duration, with an intensity initially set at 80% of VO_2_ peak on a cycle ergometer.	12	The ad libitum diet consisted of dietary macronutrient composition (based on the amount of food consumed) including 18% fat, 19% protein, 63% carbohydrate, and 26 g fiber per 1000 kcal.
Hens et al. (2021) [58]	62 (M&F)	Obese	Diet (CR) Com-Exe+Diet	VFA LF Pericardial fat Epicardial fat	CT MRI	Diet (CR): 36.11 ± 8.94 Com-Exe+Diet: 37.64 ± 8.53	Diet (CR): 32.27 ± 3.50 Com-Exe+Diet: 32.98 ± 3.60	Com-Exe 3 x/w; aerobics were at 90%–95% heart rate. Each day, core stability exercises and 4 strength exercises were added	26	A hypocaloric diet
Idoate et al. (2011) [66]	25 (F)	Obese	Diet (CR) R-Exe+Diet	VFA	MRI	Diet (CR): 51.6 ± 6.6 R-Exe+Diet (CR): 47.7 ± 6.5	Diet (CR): 34.6 ± 3.4 R-Exe+Diet (CR): 35 ± 3.1	R-Exe 2 x/w of dynamic resistance exercises for 45–60 min.	16	Hypocaloric diet: 55% of calories as carbohydrates, 15% as proteins and the rest as fat) of 500 kcal/d
Janssen et al. (2002) [67]	38 (F)	Obese	Diet A-Exe+Diet R-Exe+Diet	VFA	MRI	Diet (CR): 40.1 ± 6.7 A-Exe+Diet: 37.5 ± 6.0 R-Exe+Diet: 34.8 ± 5.8	Diet (CR): 33.7 ± 4.1 A-Exe+Diet: 36.0 ± 7.1 R-Exe+Diet: 31.6 ± 4.3	A-Exe aerobic exercise 5x/w R-Exe 3x/w.	16	For the 16-wk treatment, the subjects in all 3 groups were asked to reduce their weight maintenance energy intake by 1000 kcal/d
Larson et al. (2010) [59]	24 (M&F)	Overweight	Diet (CR) A-Exe+Diet	VFA	CT	Diet (CR): 39 ± 3.46 A-Exe+Diet: 39 ± 3.46	Diet (CR): 27.8 ± 1.038 A-Exe+Diet: 27.8 ± 1.038	A-Exe 5 d a week. Expenditure 12.5% more energy than basic needs by performing structured aerobic exercise (such as walking, running, or stationary cycling)	13	All diets were based on the American Heart Association Step 1 recommendations (≤30% fat; ≤10% saturated fat) and provided the RDA for all essential vitamins and minerals
Larson-Meyer et al. (2006) [49]	18 (M&F)	Overweight	Diet (PD) A-Exe+Diet	LF IMF VFA	CT	Diet (PD): 25–50 A-Exe and Diet: 25–50	BMI ≥ 30	A-Exe 5 x/w increased energy expenditure by 12.5% above resting by undergoing structured exercise (that is, walking, running, or stationary cycling)	26	Diet (PD) 25% calorie restriction of baseline energy requirements
Abbate et al. (2021) [68]	86 (F)	NAFLD Metabolic syndrome	Diet (CR) A-Exe+Diet	LF	MRI	Diet (CR): 52.3 ± 7.1 A-Exe+Diet^:^ 52.2 ± 5.8	Diet (CR): 34.3 ± 3.9 A-Exe Diet: 33.2 ± 3.0	The 35-min on-site training sessions were divided in 3 different phases: a 5-min warm-up, 20-min moderate intensity interval training	26	Oil, nuts, and ω-3 containing foods), 25% protein (mainly from vegetable sources), and 40%–45% carbohydrates (50%–70% of the total carbohydrate intake should low on glycemic index and rich in fiber)
Nicklas et al. (2009) [69]	96 (F)	Obese Postmenopausal	Diet (CR) A-Exe+Diet1 A-Exe+Diet_2_	VFA	VFA: CT	Diet (CR): 58.4 ± 6 A-Exe+Diet a: 57.7 ± 5.5 A-Exe+Diet b: 59 ± 5	Diet (CR): 33.9 ± 4 A-Exe+Diet_1_: 33.7 ± 3.5 A-Exe+Diet_2_: 32.9 ± 3.7	A-Exe Moderate: 3 x∖w, for 20–55 min∖session in duration with 45%–50% HRR on treadmill Vigorous: 3 x∖w, for 10–30 min∖session in duration with 70%–75% HRR on treadmill	20	The degree of caloric restriction was adjusted so that total caloric deficit (400 kcal/d; 2800 kcal/wk)
Oh et al. (2014) [73]	72 (M)	NAFLD Obese	Diet (CR) A-Exe+Diet	VFA	Ultrasonography	Diet (CR): 53.2 ± 9.39 A-Exe+Diet: 49.1 ± 9.37	Diet (CR): 28.5 ± 3.57 A-Exe+Diet: 29.2 ± 2.88	A-Exe 3 d/w of aerobics consisted of 40–60 min brisk walking and/or light jogging sessions	13	A caloric intake of 1680 kcal/d through 12 weekly lectures
Okura et al. (2007) [70]	59 (F)	Overweight Obese	Diet (CR) A-Exe+Diet	VFA	VFA: CT	Diet (CR): 52 ± 8 A-Exe+Diet: 55 ± 6	Diet (CR): 30.4 ± 4.9 A-Exe+Diet: 29.2 ± 2.3	A-Exe 3 x/w, 70%–85% MHR 45 min/session	14	First meal: 170 kcal package with protein, carbs, fat, amino acids, vitamins, minerals. Two other meals: mean 240 kcal protein, 480 kcal carbs, 240 kcal fat.
Otten et al. (2019) [71]	16 (F)	T2DM Obese Postmenopausal	Diet (PD) Com-Exe+Diet	Epicardial fat volume myocardial fat volume	Epicardial fat: MRI Myocardial fat: MRI	Diet (PD): 58.33 ± 10.33 Com-Exe: 61.66 ± 6.78	Diet (PD): 30.66 ± 0.86 Com-Exe: 32.33 ± 6.78	Com-Exe: A-Exe Frequency: 3x/w R-ex: 3x/w	12	The Paleolithic diet based on vegetables, fruits, berries, nuts, seafood, eggs, fish, and lean meat. Dairy products, cereals, legumes, refined fats, added sugar, and salt were excluded. Energy intake was *ad libitum*
Otten et al. (2018) [60]	24 (M&F)	Type 2 diabetic Obese	Diet (PD) Com-Exe+Diet	IMF IHCL	CMR	Diet (PD): 59.5 ± 2.988 Com-Exe+Diet (PD): 61.75 ± 2.683	Diet (PD): 31.47 ± 1.28 Com-Exe+Diet (PD): 31.6 ± 1.67	Com-Exe A-Exe aerobic exercise and resistance training: 3x/w. Low-intensity aerobic exercise R-Exe Resistance training of total body exercises	12	The Paleolithic diet included lean meat, eggs, fish, seafood, nuts, fruits, and vegetables. Dairy products, cereals, legumes and added sugar and salt were excluded.
Redman et al. (2010) [63]	23 (M&F)	Overweight	Diet (CR) A-Exe+Diet	VFA	VFA: CT	Diet (CR): 38.4 ± 5.30: A-Exe+Diet 35.5 ± 5.54	Diet (CR): 25–30 A-Exe+Diet: 25–30	A-Exe increased energy expenditure by 12.5% above baseline through structured aerobic exercise, 5x/w	26	25% CR from baseline energy requirements
Rice et al. (1999) [74]	29 (M)	Obese	Diet (CR) A-Exe+Diet R-Exe+Diet	VFA	MRI	Diet (CR): 44.4 ± 6.1 A-Exe+Diet: 47.4 ± 6.7 R-Exe+Diet: 39.8 ± 13.2	Diet (CR): 31.9 ± 2.8 A-Exe+Diet: 32.3 ± 3.7 R-Exe+Diet: 33.8 ± 4.2	A-Exe aerobic exercise 5x/w. R-Exe 45 min/session resistance exercise 3 x/w	16	4.18 MJ/d (1000 kcal/d)
Ross et al. (1996) [75]	33 (M)	Obese	Diet (CR) A-Exe+Diet R-Exe+Diet	VFA	MRI	Diet (CR): 46.8 ± 7.6 A-Exe+Diet: 47.6 ± 6.4 R-Exe+Diet: 39.0 ± 12.9	Diet (CR): 31.6 ± 2.7 A-Exe+Diet: 32.6 ± 3.6 R-Exe+Diet: 33.5 ± 4.1	A-Exe 5 d a week 15–60 min R-Exe Resistance training 3 d a week, 8 exercises and 8–12 repetitions with 30%–45% of RM in each session	16	Weight-maintenance energy intake was reduced by 4.19 MJ/d (1000 kcal/d).
Shah et al. (2009) [61]	18 (M&F)	Obese	Diet (CR) Com-Exe+Diet	LF	LF: H-MRS	Diet (CR): 68.6 ± 3.3 Com-Exe+Diet: 68.5 ± 3.9	Diet (CR): ≥30 Com-Exe+Diet: ≥30	Com-Exe A-Exe 70%–85% HRpeak, 90 min/w R-ex: 1–3 sets, from 8–12 reps to 6–8 reps, 65%–80% 1RM, 90 min/w Flexibility: 45 min/w	26	CR until 10% weight loss
Snel et al. (2012) [62]	27 (M&F)	T2DM Obese Postmenopausal	Diet (CR) A-Exe+Diet	IMF	IMF: Oil Red O staining	Diet (CR): 56.1 ± 8.97 A-Exe+Diet: 53 ± 9.01	Diet (CR): 37.9 ± 5.23 A-Exe+Diet: 36.4 ± 3.96	A-Exe 30 min at home, 1 h in hospital. Avg home cycle ergometer training: 5.25x/w, 35.7 min/training.	16	A total of ∼450 kcal/d and all necessary vitamins and micronutrients, divided over 3 meals of liquid shakes, ∼50 g protein, 50–60 g carbohydrate, 7–9 g lipid, and 10 g of dietary fiber
Tamura et al. (2005) [47]	14 (M&F)	Type 2 diabetic Overweight	Diet (CR) A-Exe+Diet	IMF IHCL	Proton MRS	Diet (CR): 55.0 ± 12.696 A-Exe+Diet (CR): 46.3 ± 7.406	Diet (CR): 27.4 ± 8.46 A-Exe+Diet (CR): 27.1 ± 7.67	A-Exe 2 or 3 sessions of exercise (30 min each) by walking on 5–6 x/w	2	60% carbohydrate, 25% fat, and 15% protein; mean total energy intake of 27.9 kcal/kg ideal body weight
Toledo et al. (2008) [64]	16 (M&F)	Obese	Diet (CR) A-Exe+Diet	IMF VFA	IMF: histology VFA: CT	Diet (CR): 46.1 ± 5.29 A-Exe and Diet: 42.4 ± 4.1	Diet (CR): 33.4 ± 3.17 A-Exe and Diet: 34.8 ± 3.3	A-Exe 3–5 x/w for 30–40 min at 60%–70% MHR by walking	16	25% reduction in calories
Yoshimura et al. (2014) [29]	33 (M&F)	Overweight	Diet (CR) A-Exe+Diet	VFA	CT	Diet (CR): 52 ± 8.484 A-Exe+Diet (CR): 61 ± 7.744	Diet (CR): 28.4 ± 2.42 A-Exe+Diet (CR): 27.3 ± 4.25	A-Exe 20 min step exercises, bicycle ergometry, walking/running (60 min/session), 3x/w supervised, plus 120 min unsupervised at home	12	Target energy intake was 25 kcal/kg of ideal body weight
You et al. (2004) [72]	34 (F)	Overweight Obese Postmenopausal	Diet (CR) A-Exe+Diet	VFA	VFA: CT	Diet (CR): 57 ± 4.12 A-Exe+Diet: 59 ± 4.12	Diet (CR): 25–40 A-Exe+Diet: 25–40	A-Exe 3 x/w, for 20–60 min/session in duration with 50%–70% HRR on treadmill	26	A hypocaloric diet designed to elicit a 0.5- to 1.0-kg weight loss/wk (∼250–350 kcal/d deficit)

Abbreviations: A-Exe, aerobic exercise; Com-Exe, combined exercise; CR, calorie restriction; CT, computed tomography; Exe, exercise; F, female; HIIT, high-intensity interval training; HRpeak, peak rate of heart rate; HRR, heart rate reserve; IHCL, intra-hepatocellular lipid; IMAT, intramuscular adipose tissue; IMF, Intramuscular fat; LCD, low carbohydrate diet; LF, liver fat; LFD, low-fat diet; M, man; Med diet, Mediterranean diet; MD, mediterranean diet; MHR, maximum heart rate; MRS, magnetic resonance spectroscopy; MUFA, monounsaturated fat; NAFLD, nonalcoholic fatty liver disease; PD, Paleolithic diet; RDA, recommended dietary allowance; R-Exe, resistance exercise; RM, repetition maximum; SAFA, saturated fatty acid; T2DM, type 2 diabetes mellitus; VFA, visceral fat area; VO2peak, peak rate of oxygen consumption; VO2max, max rate of oxygen consumption; WK, weak; x, day.

### Intervention characteristics

Intervention durations ranged from 2 [47] to 78 [48] wk, with 26-wk durations in the majority of studies [22,49,50,53,55,56,58,61,63,68,72]. In 4 studies, resistance training and diet were compared with a diet [66,67, 74,75], 23 studies compared aerobic training and diet compared with diet [29,30,47,[49], [50], [51],[53], [54], [55], [56], [57],^59^,[62], [63], [64], [65],[67], [68], [69], [70],[72], [73], [74], [75]], and 7 studies compared combined training and diet compared with diet [48,22,52,58,60,61,71]. Five studies used >1 type of exercise protocol as separate interventions [65,67,69,74,75]. Exercise sessions were performed 2 [22] to 6 [47] times per week, with 3 sessions being the most common.

The duration of each session of resistance training ranged from 30 min [74] to 60 min [66]. The intensity of each session of resistance training ranged from 30% 1-repetition maximum (1RM) [75] to 45% 1RM [75].

The duration of each session of aerobic training ranged from 15 min [65] to 60 min [72]. Also, the intensity of each session of aerobic training ranged from 50% to 85% maximum heart rate (MHR).

The duration of each session of combined training varied from 30 min [48] to 70 min [71]. The intensity of each session of resistance training ranged from 65% 1RM [61] to 80% 1RM [61], and the intensity of each session of aerobic training ranged from 65% MHR [48] to 95% MHR [58].

Most included studies (26 out of 32) used CR [22,29,47,50,51,53,[54], [55], [56], [57], [58], [59],[61], [62], [63], [64],[65], [66], [67], [68], [69], [70],[72], [73], [74], [75]]; 4 studies used the PD [49,52,60,71], 1 study used alternate-day fasting [30], and 1 study used the Mediterranean diet [48]. The PD is a modern fad diet containing vegetables, fruits, nuts, roots, and meat and excludes dairy products, sugar, salt, legumes, processed oils, grains, alcohol, and coffee. The characteristics of intervention are presented in Table 1.

### Meta-analysis

#### Exercise and diet compared with diet only

##### Body weight

**FIGURE 2:**
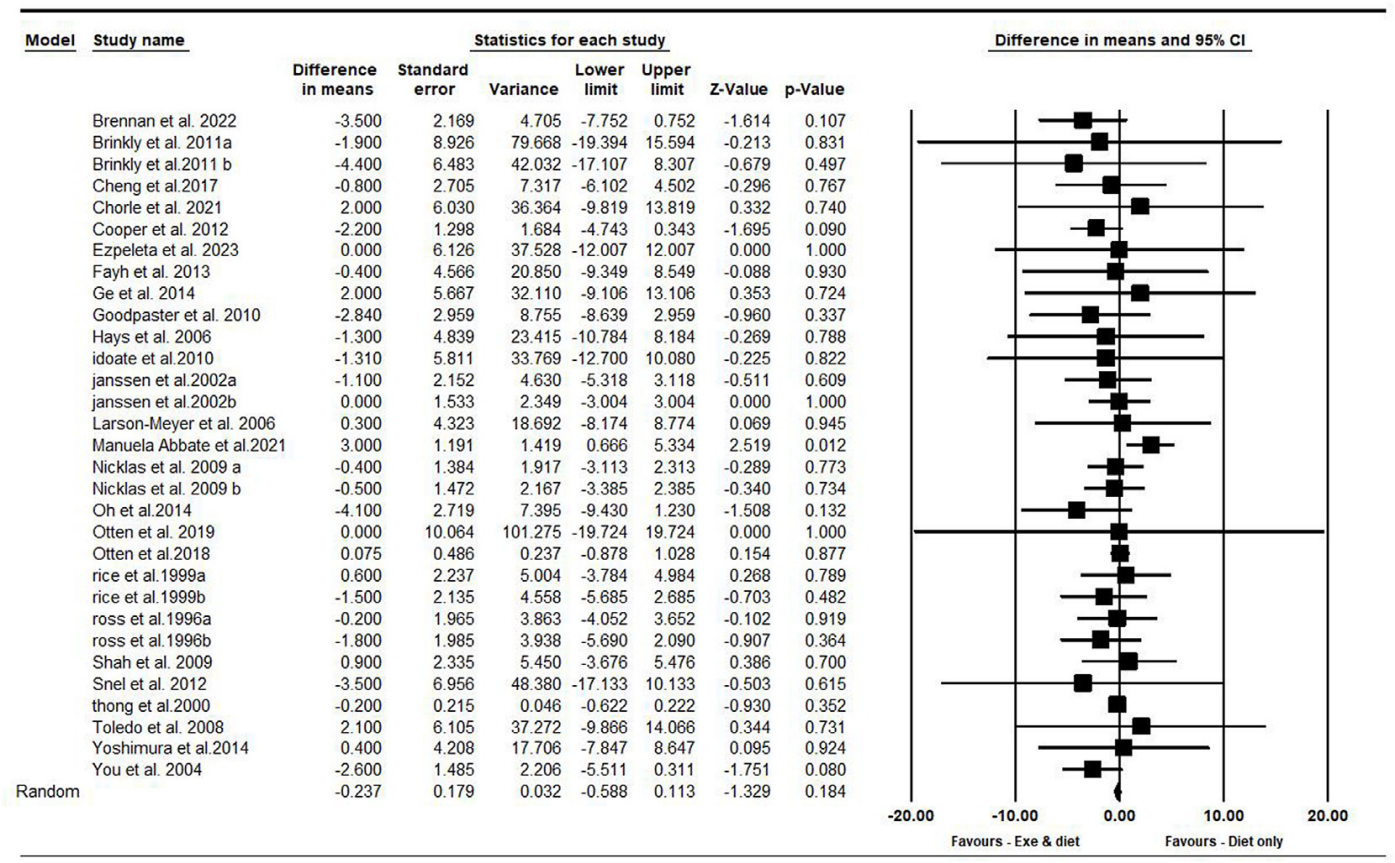
Forest plot of the effects of exercise and diet compared with diet only on body weight. Data are reported as WMD (kg) (95% confidence limits). WMD, weighted mean difference.

On the basis of 31 intervention arms with 1081 participants, exercise and diet did not change body weight [WMD = –0.23 kg (95% CI: –0.58, 0.11), *P* = 0.180] when compared with a diet-only group (Figure 2). There was no significant heterogeneity among included studies (*I*^2^ = 0.00%, *P* = 0.900). Visual interpretation of funnel plots and Egger’s test (*P* = 0.200) results also did not show publication bias. Sensitivity analysis performed by removing individual studies showed that the significance of results and direction of the results did not change.

Subgroup analyses by health status revealed no significant reductions in body weight for adults without [WMD = –0.27 kg (95% CI: –0.67, 0.12), *P* = 0.170, 15 interventions] or with disease [WMD = –0.16 kg (95% CI: –1.01, 0.67), *P* = 0.690, 16 interventions] when compared with a diet-only group.

In addition, subgroup analyses by type of exercise revealed no significant reductions in body weight for aerobic [WMD = –0.24 kg (95% CI: –0.63, 0.13), *P* = 0.200, 22 interventions], combined [WMD = –0.04 (95% CI: –0.95, 0.86), *P* = 0.920, 5 interventions], or resistance exercises [WMD = –0.88 kg (95% CI: –2.92, 1.14), *P* = 0.390, 4 interventions] when compared with a diet-only group.

In addition, subgroup analyses by intervention duration revealed no significant reductions in body weight for long term >12 wk [WMD = –0.64 kg (95% CI: –1.50, 0.21), *P* = 0.140, 20 interventions], or short-term intervention ≤12 wk [WMD = –0.15 kg (95% CI: –0.53, 0.22), *P* = 0.420, 11 interventions] when compared with a diet-only group.

In addition, subgroup analyses by BMI indicated no significant reductions in body weight for participants with obesity [WMD = –0.20 kg (95% CI: –0.55, 0.15), *P* = 0.250, 26 interventions], with overweight [WMD = 0.35 kg (95% CI: –5.55, 6.26), *P* = 0.900, 2 interventions], or obesity and overweight [WMD = –2.30 kg (95% CI: –5.12, 0.51), *P* = 0.100, 2 interventions] when compared with a diet-only group.

In addition, subgroup analyses by type of diet indicated no significant reductions in body weight for participants with calorie restriction [WMD = –0.37 kg (95% CI: –0.75, 0.001), *P* = 0.050, 26 interventions], or with PD [WMD = 0.08 kg (95% CI: –0.86, 1.03), *P* = 0.850, 3 interventions] when compared with a diet-only group.

In addition, subgroup analyses by gender indicated no significant reduction in body weight for males [WMD = –0.24 kg (95% CI: –0.65, 0.16), *P* = 0.240, 6 interventions], females [WMD = –0.89 kg (95% CI: –2.22, 0.42), *P* = 0.180, 10 interventions], or males and females [WMD = 0.01 kg (95% CI: –0.75, 0.78), *P* = 0.960, 15 interventions] when compared with a diet-only group.

##### Liver fat

**FIGURE 3:**
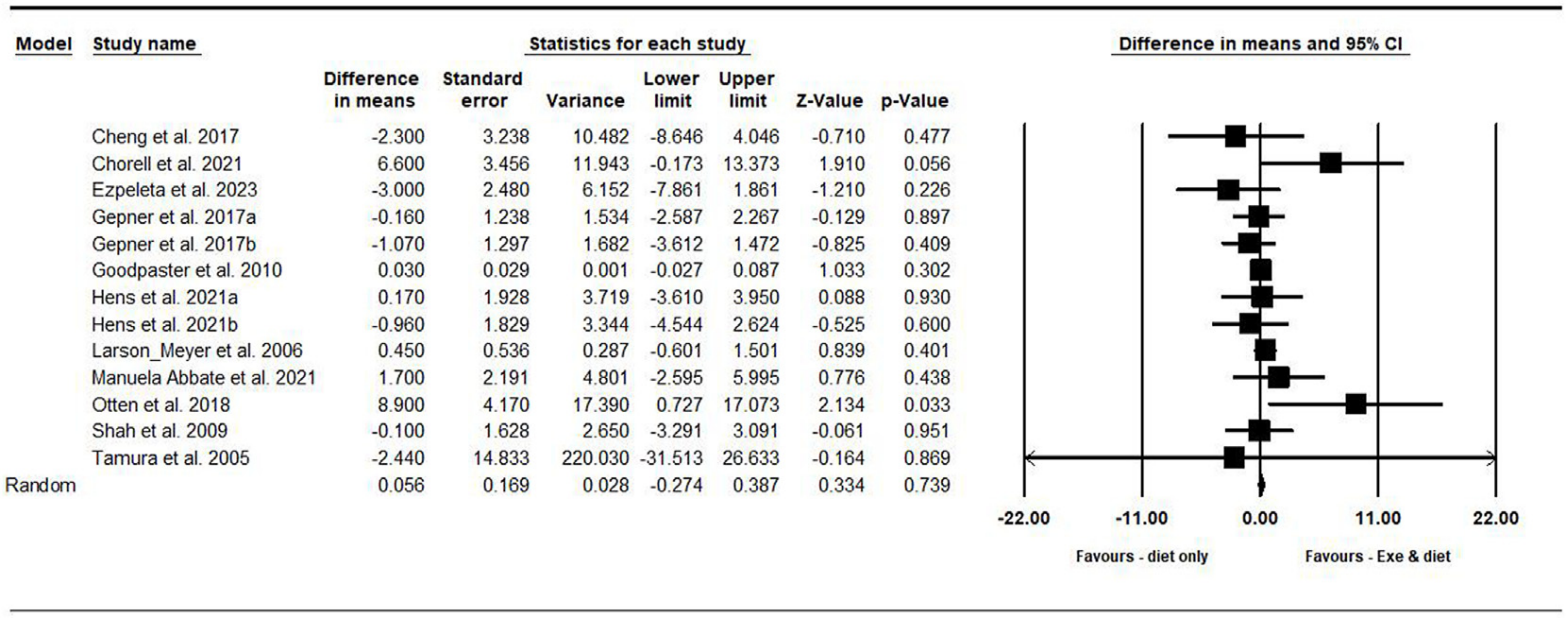
Forest plot of the effects of exercise and diet compared with diet only on liver fat. Data are reported as WMD (percent) [95% confidence limits (CI)]. WMD, weighted mean difference.

On the basis of 13 intervention arms with 767 participants, exercise and diet did not change liver fat [WMD = 0.05% (95% CI: –0.27, 0.38), *P* = 0.730], when compared with a diet-only group (Figure 3). There was no significant heterogeneity among the included studies (*I*^2^ = 3.36%, *P* = 0.410). Visual interpretation of funnel plots and Egger’s test (*P* = 0.600) results did not show publication bias. Sensitivity analysis performed by removing individual studies showed that the significance of results and direction of the results did not change.

Subgroup analyses by health status revealed no significant change in liver fat for adults without disease [WMD = 0.03% (95% CI: –0.02, 0.08), *P* = 0.280, 3 interventions] or with disease [WMD = 0.04% (95% CI: –1.51, 1.59), *P* = 0.950, 10 interventions] when compared with a diet-only group.

Subgroup analyses by type of exercise revealed no significant change in liver fat for aerobic [WMD = 0.03% (95% CI: –0.02, 0.08), *P* = 0.290, 8 interventions] or combined exercises [WMD = 0.95% (95% CI: –1.52, 3.44), *P* = 0.440, 5 interventions] when compared with a diet-only group.

Subgroup analyses by intervention duration revealed no significant change in liver fat for long-term >12 wk [WMD = 0.03% (95% CI: –0.02, 0.08), *P* = 0.290, 8 interventions] or short-term interventions ≤12 wk [WMD = 2.11% (95% CI: –2.39, 6.62), *P* = 0.350, 5 interventions] when compared with a diet-only group.

Subgroup analyses by BMI revealed no significant change in liver fat for participants with obesity [WMD = 0.02% (95% CI: –0.87, 0.93), *P* = 0.950, 10 interventions] or with normal weight [WMD = –2.30% (95% CI: –8.50, 3.89), *P* = 0.460, 2 interventions] when compared with a diet-only group.

Because of the small number of studies, it is not possible to perform subgroup analysis based on the type of diet.

Subgroup analyses by gender status revealed no significant change in liver fat for males [WMD = –0.59% (95% CI: –2.34, 1.16), *P* = 0.500, 2 interventions] or males and females [WMD = 0.16% (95% CI: –0.46, 0.79), *P* = 0.600, 11 interventions] when compared with a diet-only group.

Metaregression determined whether or not body weight loss influenced the effects of exercise and diet on liver fat, in which no significant correlation was found (coefficient: –0.06; 95% CI: –0.60, 0.48, *P* = 0.820). This result suggested that there was no significant moderator effect on body weight loss.

##### Visceral fat area

**FIGURE 4:**
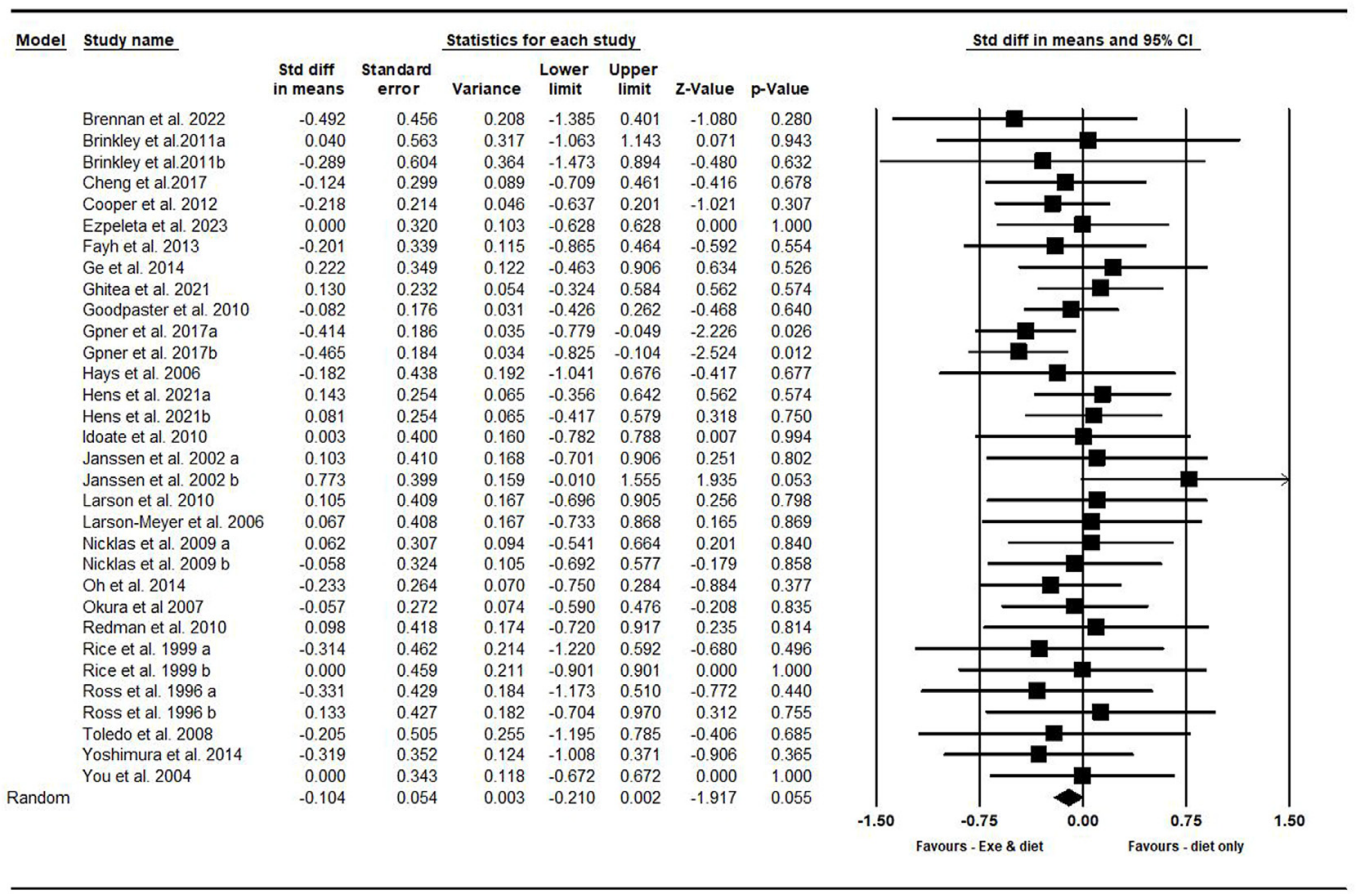
Forest plot of the effects of exercise and diet compared with diet only on VFA. Data are reported as SMD [95% confidence limits (CI)]. SMD, standardized mean difference; VFA, visceral fat area.

On the basis of 32 intervention arms with 1487 participants, exercise and diet did not significantly reduce VFA [SMD = –0.10 (95% CI: –0.21, 0.001), *P* = 0.050] when compared with a diet-only group (Figure 4). There was no significant heterogeneity among the included studies (*I*^2^ = 0.00%, *P* = 0.950). Visual interpretation of funnel plots and Egger’s test (*P* = 0.080) results also did not show publication bias.

Sensitivity analysis performed by removing individual studies revealed that removing Ge et al. [50], Ghitea et al. [55], Hens et al. [27], Hens et al. [58], Janssen et al. [67], Larson et al. [59], Nicklas et al. [69], and Ross et al. [75], studies resulted in a change in the effect size and significance (SMD = –0.11%, *P* = 0.040), (SMD = –0.11%, *P* = 0.030), (SMD = –0.11%, *P* = 0.030), (SMD = –0.11%, *P* = 0.040), (SMD = –0.10%, *P* = 0.040), (SMD = –0.12%, *P* = 0.020), (SMD = –0.10%, *P* = 0.040), (SMD = –0.10%, *P* = 0.040), and (SMD = –0.10%, *P* = 0.040), respectively, whereas the direction of the results remained consistent.

Subgroup analyses by health status revealed no significant reductions in VFA for adults without disease [SMD = –0.06 (95% CI: –0.22, 0.10), *P* = 0.460, 16 interventions] or with disease [SMD = –0.13 (95% CI: –0.27, 0.004), *P* = 0.050, 16 interventions] when compared with a diet-only group.

In addition, subgroup analyses by type of exercise revealed no significant reductions in VFA for aerobic [SMD = –0.04 (95% CI: –0.17, 0.08), *P* = 0.530, 25 interventions] or combined exercises [SMD = –0.22 (95% CI: –0.45, 0.00), *P* = 0.050, 6 interventions] when compared with a diet-only group.

In addition, subgroup analyses by intervention duration revealed no significant differences in VFA for long term >12 wk [SMD = –0.11 (95% CI: –0.23, 0.00), *P* = 0.050, 26 interventions] or short-term interventions ≤12 wk [SMD = –0.04 (95% CI: –0.30, 0.21), *P* = 0.740, 6 interventions] when compared with a diet-only group.

In addition, subgroup analyses by BMI indicated significant reduction in VFA for participants with obesity [SMD = –0.12 (95% CI: –0.24, –0.006), *P* = 0.040, 24 interventions] but not for those with overweight [SMD = –0.03 (95% CI: –0.42, 0.34), *P* = 0.840, 4 interventions] or with overweight and obesity [SMD=0.03 (95% CI: –0.32, 0.39), *P* = 0.840, 3 interventions] when compared with a diet-only group.

Because the studies were not included in the 2 categories of caloric restriction or PD, subgroup analysis was not performed based on the type of diet.

In addition, subgroup analyses by gender indicated a significant decrease in VFA for males [SMD = –0.33 (95% CI: –0.54, –0.13), *P* = 0.001, 7 interventions] but not for females [SMD = 0.08 (95% CI: –0.14, 0.30), *P* = 0.490, 10 interventions] or males and females [SMD = –0.05 (95% CI: –0.20, 0.09), *P* = 0.450, 15 interventions] when compared with a diet-only group.

Metaregression determined whether or not body weight loss influenced the effects of exercise and diet on VFA, in which no significant correlation was found (coefficient: –0.002; 95% CI: –0.04, 0.03; *P* = 0.910). This result suggested that there was no significant moderator effect on body weight loss.

##### Intramuscular fat

**FIGURE 5:**
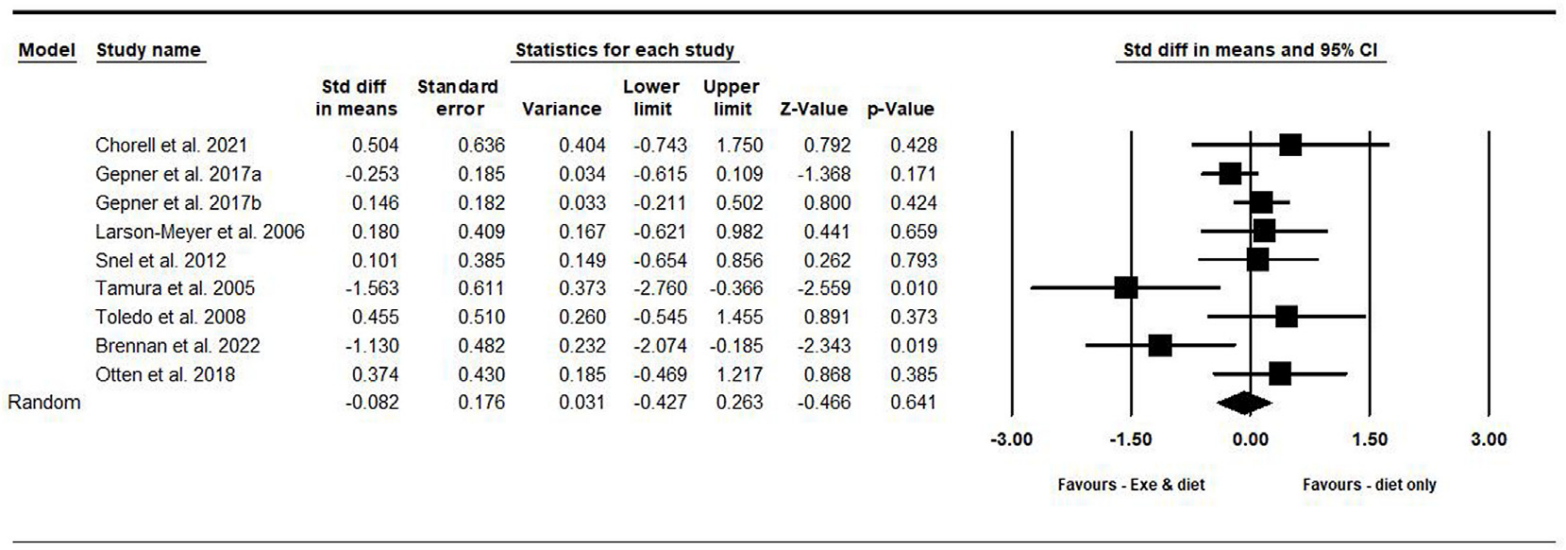
Forest plot of the effects of exercise and diet compared with diet only on intramuscular fat. Data are reported as SMD [95% confidence limits (CI)]. SMD, standardized mean difference.

On the basis of 9 intervention arms with 375 participants, exercise and diet did not significantly change IMF [SMD = –0.08 (95% CI: –0.42, 0.26), *P* = 0.640] when compared with a diet-only group (Figure 5). There was significant heterogeneity among included studies (*I*^2^ = 52.05%, *P* = 0.030). Visual interpretation of funnel plots and Egger’s test (*P* = 0.730) results also did not show publication bias. Sensitivity analysis performed by removing individual studies showed that the significance of results and direction of the results did not change.

Subgroup analyses by health status revealed no significant differences in IMF for adults without disease [SMD = 0.28 (95% CI: –0.33, 0.91), *P* = 0.36, 2 interventions] or with disease [SMD = –0.17 (95% CI: –0.58, 0.23), *P* = 0.390, 7 interventions] when compared with a diet-only group.

Subgroup analyses by type of exercise revealed no significant differences in IMF for aerobic [SMD = –0.12 (95% CI: –0.85, 0.60), *P* = 0.740, 4 interventions] or combined exercise [SMD = –0.08 (95% CI: –0.49, 0.33), *P* = 0.700, 5 interventions] when compared with a diet-only group.

In addition, subgroup analyses by intervention duration revealed no significant differences in IMF for long-term >12 wk [SMD = –0.06 (95% CI: –0.39, 0.26), *P* = 0.710, 6 interventions] or short-term intervention ≤12 wk [SMD = –0.20 (95% CI: –1.44, 1.04), *P* = 0.750, 3 interventions] when compared with a diet-only group.

Because the studies were not included in the 2 categories of obesity or overweight, subgroup analysis was not performed based on BMI.

In addition, subgroup analyses by type of diet indicated no significant differences in IMF for participants with calorie restriction [SMD = –0.28 (95% CI: –0.77, 0.19), *P* = 0.240, 6 interventions] or with PD [SMD = 0.41 (95% CI: –0.28, 1.11), *P* = 0.240, 2 interventions] when compared with a diet-only group.

In addition, subgroup analyses by gender indicated no significant differences in IMF fat for males [SMD = –0.05 (95% CI: –0.44, 0.33), *P* = 0.793, 2 interventions] or males and females [SMD = –0.11 (95% CI: –0.67, 0.43), *P* = 0.672, 7 interventions] when compared with a diet-only group.

Metaregression determined whether or not body weight loss influenced the effects of exercise and diet on IMF, in which no significant correlation was found (coefficient: 0.005; 95% CI: –0.03, 0.05; *P* = 0.800). This result suggested that there was no significant moderator effect on body weight loss.

##### Pericardial and epicardial fat

**FIGURE 6:**
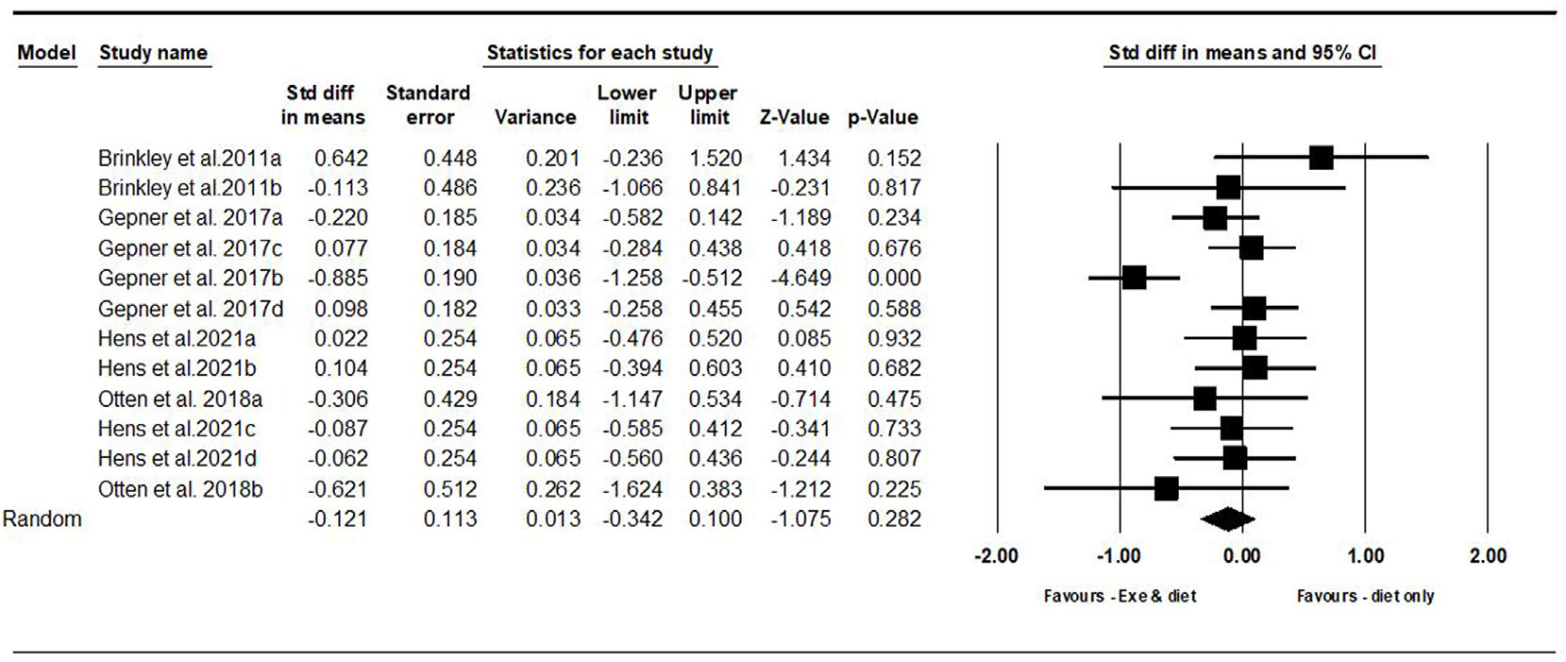
Forest plot of the effects of exercise and diet compared with diet only on pericardial & epicardial fats. Data are reported as SMD [95% confidence limits (CI)]. SMD, standardized mean difference.

On the basis of 12 intervention arms with 375 participants, exercise combined with diet did not significantly change pericardial and epicardial fats [SMD = –0.12 (95% CI: –0.34, 0.10), *P* = 0.280] when compared with a diet-only group (Figure 6). There was significant heterogeneity among included studies (*I*^2^ = 54.47%, *P* = 0.010). Visual interpretation of funnel plots and Egger’s test (*P* = 0.710) results also did not show publication bias. Sensitivity analysis performed by removing individual studies showed that the significance of results and direction of the results did not change.

Because the studies were not included in the 2 categories of adults with disease or without disease, subgroup analysis was not performed based on the health status.

Subgroup analyses by type of exercise revealed no significant differences in pericardial and epicardial fats for aerobic [SMD = –0.02 (95% CI: –0.23, 0.19), *P* = 0.850, 8 interventions] or combined exercises [SMD = –0.23 (95% CI: –0.67, 0.21), *P* = 0.300, 4 interventions] when compared with a diet-only group.

In addition, subgroup analyses by intervention duration revealed no significant differences in pericardial and epicardial fats for long-term >12 wk [SMD = –0.15 (95% CI: –0.47, 0.16), *P* = 0.340, 6 interventions] or short-term interventions ≤12 wk [SMD = –0.04 (95% CI: –0.32, 0.23), *P* = 0.740, 6 interventions] when compared with a diet-only group.

Because the studies were not included in the 2 categories of adults with obesity or overweight, subgroup analysis was not performed based on the BMI.

In addition, subgroup analyses by type of diet indicated no significant differences in pericardial and epicardial fats for participants following calorie restriction [SMD = 0.04 (95% CI: –0.14, 0.24), *P* = 0.640, 8 interventions], those following PD [SMD = –0.43 (95% CI: –1.08, 0.20), *P* = 0.180, 2 interventions], and participants following Mediterranean diet [SMD = –0.39 (95% CI: –1.35, 0.57), *P* = 0.420, 2 interventions] when compared with a diet-only group.

In addition, subgroup analyses by gender indicated no significant differences in pericardial and epicardial fats for males [SMD = –0.23 (95% CI: –0.67, 0.21), *P* = 0.300, 4 interventions], females [SMD = 0.28 (95% CI: –0.45, 1.02), *P* = 0.440, 2 interventions], or males and females [SMD = –0.06 (95% CI: –0.29, 0.17), *P* = 0.600, 6 interventions] when compared with a diet-only group.

Metaregression determined whether or not body weight loss influenced the effects of exercise and diet on pericardial and epicardial fats, in which no significant correlation was found (coefficient: –0.07; 95% CI: –0.20, 0.04; *P* = 0.210). This result suggested that there was no significant moderator effect of body weight loss.

##### Pancreatic fat

On the basis of 2 intervention arms with 240 participants, exercise combined with diet revealed no significant differences in pancreatic fat [WMD = –0.24% (95% CI: –0.79, 0.29), *P* = 0.370], when compared with a diet-only group (Supplemental Figure 1). There was no significant heterogeneity among included studies (*I*^2^ = 0.00%, *P* = 0.900).

##### Renal sinus fat

On the basis of 2 intervention arms with 240 participants, exercise combined with diet revealed no significant differences in renal sinus fat [WMD = 0.01 cm^2^ (95% CI: –0.004, 0.02), *P* = 0.170], when compared with a diet-only group (Supplemental Figure 2). There was significant heterogeneity among the included studies (*I*^2^ = 0.00%, *P* = 0.740).

### Quality assessment

The methodological quality of individual studies was evaluated using the PEDro tool with scores ranging from 6 to 9 out of a maximum of 9 points. Three studies had scores of 9, 8 studies had scores of 8, 13 studies had scores of 7, and 8 scored 6. Most of the studies received lower scores because of 3 evaluation criteria (concealed allocation, blinding of all assessors, and outcome measures assessed in 85% of participants). The details of the quality of studies are provided in Supplemental Table 1.

## Discussion

To assess the synergistic impacts of dietary intervention and physical activity intervention compared with dietary intervention alone on ectopic lipid accumulation in individuals classified as overweight and obese, we performed a meta-analysis encompassing 32 controlled investigations. According to our findings, exercise in conjunction with dietary interventions significantly reduces ectopic fat storage in both populations with overweight and obesity, including visceral fat, IMF, IHL, pericardial and epicardial fat, pancreatic fat, and renal sinus fat, when compared with diet alone. Regarding the classifications of exercise modalities, we conducted a subgroup analysis, categorizing the studies into 3 distinct groups: aerobic exercise, resistance training, and combined exercise. The findings of the subgroup analysis indicated that the patterns of the impacts of these 3 forms of exercise on body weight and excess adipose tissue are predominantly consistent. Furthermore, with respect to dietary approaches, we executed a subgroup analysis, dividing the studies into 2 categories: caloric restriction and dietary maintenance. The outcomes of the subgroup analysis revealed that the patterns of the impacts of these 2 dietary strategies on body weight and excess adipose tissue are mainly stable. The analysis of subgroups predicated on the length of the intervention and the health condition of the subjects did not yield statistically significant findings. However, it is interesting to note that, compared with diet alone, dietary interventions plus exercise significantly decreased VFA in patients with obesity, according to subgroup analysis based on BMI. Additionally, exercise and nutritional changes dramatically decreased VFA in males but not females. These 2 findings hold significant relevance as they demonstrate the susceptibility of individuals with obesity, both male and female, to interventions involving dietary modifications and physical activity aimed at diminishing visceral fat accumulation.

To date, numerous studies have demonstrated that although calorie restriction can result in immediate body weight reduction, interventions incorporating both nutritional changes and physical activity prove more effective in achieving sustained long-term body weight loss outcomes [32,76,77]. However, the results of this study showed that when compared with diet alone, none of the interventions are superior for body weight loss, despite the lack of heterogeneity and publication bias in these studies. However, it is crucial to acknowledge that body weight loss alone does not necessarily reflect improvements in overall health, underscoring the significance of assessing changes in body composition following both interventions [78]. The findings show no distinction in the reduction of IMF between diet-only and diet-plus-exercise regimens. Importantly, evidence suggests that diet composition profoundly influences intramuscular triglyceride (IMTG) levels, potentially modulating IMTG response to training [79]. For instance, diets high in saturated fats are associated with greater IMTG accumulation and lipid droplet size, which can lead to impaired insulin sensitivity due to increased lipotoxic intermediates, such as ceramides and diacylglycerols [80]. In contrast, polyunsaturated fats may mitigate IMTG accumulation, as they enhance lipid oxidation and reduce the synthesis of harmful lipid metabolites [81]. Furthermore, IMF includes a mix of triglycerides, phospholipids, and cholesterol esters stored between muscle fibers rather than inside muscle cells [82]. Because of this, IMF may respond differently to diet and exercise than IMTG, which is more metabolically active and readily used as fuel during exercise. A study by Goodpaster et al. [83] suggested that IMF can serve as a more stable energy reserve, and is not as readily mobilized as IMTG, especially in response to moderate dietary or exercise interventions. This stability could explain why the IMF levels remained unchanged despite exercise. It is important to acknowledge that an individual’s physical fitness level may influence variations in the proportion and metabolism of IMF. To illustrate, in the phenomenon commonly referred to as the “athlete’s paradox,” elevated levels of IMF are observed in well-conditioned endurance athletes, attributable to physiological adaptations resulting from rigorous training [84]; this investigation did not consider the participants’ physical fitness levels. Given that IMF plays a crucial role in mediating metabolic and endocrine functions [85,86], evaluating the impact of dietary intake and physical activity on IMF concentrations is of significant clinical relevance.

Aerobic-based exercise, including high-intensity interval training, is often promoted as the most effective exercise mode for reducing total fat mass, as well as improving cardiorespiratory fitness and insulin sensitivity [87]. However, previous meta-analyses have demonstrated that the incorporation of aerobic or resistance training within an energy-restricted dietary regimen does not enhance the reduction of visceral adiposity [67,88]. Our results support these findings by showing that neither intervention was more effective in lowering VFA, even in the absence of heterogeneity and publication bias. However, other studies have reported varying outcomes on this topic. For instance, Verheggen et al. [89] conducted a systematic review and meta-analysis and found that exercise training, particularly aerobic exercise, significantly reduces VAT, independent of weight loss. This suggests that exercise might have direct effects on visceral fat metabolism beyond caloric expenditure [89]. Similarly, Ohkawara et al. [88] reported that the dose–response relationship between aerobic exercise and visceral fat reduction indicates that higher volumes of exercise lead to greater reductions in visceral fat. Conversely, another study found that although physical activity contributes to abdominal fat reduction, combining diet with exercise did not produce significantly greater losses in visceral fat compared with diet alone in some cases [90]. Ross et al. [91] also observed that diet-induced weight loss resulted in similar reductions in visceral fat as exercise-induced weight loss, emphasizing the role of negative energy balance regardless of the method. These conflicting findings highlight the complexity of visceral fat reduction and suggest that factors such as exercise intensity, duration, and individual metabolic responses may influence outcomes. It is possible that the nonsignificant findings in our meta-analysis could be due to variations in these factors among the included studies. Although both exercise and dietary restriction yielded comparable effects on the reduction of visceral fat in our analysis, it is crucial to recognize the additional advantages of exercise on various health outcomes. Exercise enhances glucose metabolism, optimizes lipoprotein profiles, and improves insulin sensitivity [[92], [93], [94]]. Slentz et al. demonstrated that aerobic exercise improves insulin action and reduces metabolic syndrome risk factors, even without significant weight loss [95]. Furthermore, Ross et al. [91] emphasized that exercise-induced improvements in cardiometabolic health can occur independently of changes in body composition [95]. Therefore, even if the addition of exercise to dietary interventions does not significantly enhance visceral fat reduction compared with diet alone in some studies, the holistic health benefits associated with physical activity seemingly justify its inclusion in obesity management strategies.

When examining exercise interventions alongside dietary changes compared with diet alone, multivariate metaregression analysis revealed that individuals with obesity, compared with individuals with overweight, as well as males, as opposed to females, experienced a greater decrease in VFA. The reduction in VFA observed in individuals with obesity, despite the lack of a significant correlation with weight loss, could be explained by several factors. First, exercise and dietary interventions often lead to body composition changes, including fat loss and lean mass preservation/increase, which may not be reflected in total body weight. Second, VAT is more metabolically active and responsive to interventions than subcutaneous fat, allowing for site-specific fat loss independent of overall weight changes [96]. Third, participants with higher baseline levels of visceral fat, such as those with obesity, may experience greater reductions in VFA compared with those with lower baseline levels, resulting in more detectable changes. Finally, the absence of a significant correlation in the metaregression may reflect heterogeneity across studies in protocols, populations, and designs, which could obscure the relationship between weight loss and VFA reduction. These findings underscore the importance of assessing VFA as a distinct outcome to better capture the metabolic benefits of exercise and diet.

It should be noted that the discrepancy in VFA reduction between males and females can be attributed to variations in hormonal profiles and body composition. Males typically exhibit higher levels of testosterone, which facilitates the breakdown of visceral fat, resulting in greater reductions compared with females [97,98]. Furthermore, males commonly exhibit greater muscle mass relative to females [99,100], potentially resulting in a higher metabolic rate and enhanced capacity for fat burning [101]. Furthermore, males often have an “android” fat distribution, characterized by fat accumulation in the abdominal region, including visceral fat. Conversely, females have a “gynoid” fat distribution, characterized by increased fat accumulation in the hips and thighs [102]. Visceral fat has more metabolic activity, facilitating its breakdown relative to subcutaneous fat [72]. Males may also have a more efficient response to exercise when mobilizing and burning visceral fat with males being shown to lose more visceral fat during exercise than females [103]. It is important to note that most of the females involved in our studies were in menopause. Given that the increase in visceral fat often occurs with the onset of menopause, this may explain the lack of significant reductions in visceral fat among females in the interventions mentioned [104]. The findings of this meta-analysis align with established international guidelines for obesity management, highlighting that changes in overall body weight may not always correlate with changes in VAT [89]. This can be attributed to the fact that because variation in body fat distribution among individuals is a crucial factor influencing this observation [[105], [106], [107]] when people lose body weight, they may lose a combination of visceral fat, subcutaneous fat, and muscle mass [108]. The rate of fat loss can be impacted by components such as hereditary qualities [109], age [110], hormonal adjustments [111], and lifestyle habits [112]. From a different perspective, the use of abdominal CT is considered the “reference standard” among available imaging methods for accurately determining VAT levels [113]. It should be noted that some studies included in the meta-analysis may not have utilized this imaging technique, potentially impacting the accuracy of the reported results. Therefore, it is essential to conduct more clinical studies incorporating abdominal CT scans to enhance the precision and reliability of findings related to VAT reduction interventions.

Given that no pharmacological therapies or surgical interventions have received endorsement for the management of nonalcoholic fatty liver disease, lifestyle modification, particularly through dietary and exercise interventions, serves as the fundamental strategy in the treatment of individuals afflicted with this condition [[114], [115], [116]]. Consequently, it is imperative to ascertain the most effective methods for reducing hepatic fat. Notwithstanding the lack of heterogeneity and publication bias, the present findings showed that diet combined with exercise does not significantly differ from diet alone in terms of lowering liver fat. The findings of our investigation further indicated that neither of the 2 interventions demonstrates a marked superiority over the other in the mitigation of renal adiposity. The adipose tissue located within the renal sinus may adversely affect renal function and contribute to the pathogenesis of chronic kidney disease [117]. Consequently, it is critically important for disease prophylaxis that interventions to reduce renal sinus fat through weight reduction are feasible.

Diet combined with exercise did not differ from diet alone in terms of lowering pericardial and epicardial adipose tissue (EAT) in the present study. EAT, the adipose layer situated between the parietal and visceral pericardium, provides essential functions for the myocardium, including acting as a reservoir for free fatty acids (FFAs), which supply energy to cardiomyocytes and protect coronary arteries [10]. However, excess EAT has been associated with lipotoxicity, contributing to cardiac hypertrophy and diastolic dysfunction due to an oversupply of FFAs [10]. Several studies support this, showing that high levels of EAT are linked to increased inflammation and fibrosis, particularly in individuals with type 2 diabetes [118,119] and coronary artery disease [120,121].

Additionally, pericardial and epicardial fat reduction was not modulated by body weight loss. Daily activity levels in adults are linked, independent of BMI, to pericardial fat in healthy participants, according to Hamer et al. [122]. This finding emphasizes the critical roles that exercise and sedentary lifestyles play in the deposition of EAT. The important point is that the reduction of EAT is also influenced by the type of exercise intervention. It has been shown that high-intensity interval training interventions reduced EAT volume by 5%, whereas endurance exercise interventions have demonstrated significant reductions in EAT volume, ranging from 5% to 32% [123]. Nevertheless, there was no discernible difference in the reduction of EAT between aerobic and combined exercise interventions in the current meta-analysis. The heterogeneity of the included studies could explain these contradicting results. There isn't currently agreement on any particular dietary approaches that work well to enhance the dynamics of pericardial fat depots. Furthermore, it is still unknown how modifications in pericardial fat tissues during dietary weight loss interventions relate to changes in hemodynamic and cardiometabolic profiles [123], which can be the subject of future studies. Research also highlights the challenges of measuring EAT across studies. Although echocardiography is commonly used due to accessibility, Iacobellis et al. [124] demonstrated that MRI provides more precise EAT quantification, revealing stronger associations between EAT volume and metabolic syndrome markers than echocardiography. Variations in measurement methods might explain the discrepancies in findings across studies, as well as differences in intervention protocols.

The meta-analysis findings suggest no significant difference between diet alone and diet combined with exercise in reducing pancreatic fat. Pancreatic fat is particularly challenging to assess because of the pancreas’s irregular size and shape, which can result in variability in fat quantification across studies [125]. Research on pancreatic fat and lifestyle interventions remain limited [126]. Van der Zijl et al. [127] found that although calorie restriction combined with exercise reduces liver fat significantly, it had a minimal effect on pancreatic fat in individuals with prediabetes, suggesting that pancreatic fat may be less responsive to the general caloric deficit. Another study noted that pancreatic fat is more closely associated with dietary patterns that promote insulin resistance, such as diets high in refined carbohydrates and low in fiber, rather than overall fat content [128]. This suggests that pancreatic fat reduction may require targeted dietary approaches that address insulin sensitivity.

Our study’s dependence on self-reported dietary assessments, which are known to have inherent limitations, is one of its limitations. Furthermore, although it is known that increasing exercise volume generally leads to a reduction in body fat, it is still unknown what level of intensity is ideal for achieving these benefits in relation to a given caloric intake. Because of the few eligible studies, our meta-analysis was limited in its ability to perform subgroup analyses to ascertain effect sizes across various exercise types, intensities, or durations. To further understand the effects of different exercise parameters on outcomes, such as type, intensity, and duration, more research is necessary. The limited number of studies included in the subgroup analyses prevents drawing definitive conclusions about the influence of specific diet types on the observed outcomes, underscoring the need for further research with more comprehensive data on dietary composition. Also, the lack of detailed reporting on participants’ ethnic backgrounds in the included studies limited our ability to perform subgroup analyses based on ethnicity, highlighting the need for future research to explore the influence of ethnicity on the outcomes of interest.

In conclusion, in summation, the present meta-analysis offers empirical evidence indicating that interventions encompassing exercise and dietary modifications do not surpass the effectiveness of dietary interventions alone in diminishing adiposity among individuals classified as obese or overweight. Nevertheless, compared with dietary interventions alone, the integration of exercise with dietary modifications resulted in a statistically significant reduction in VFA among participants with obesity, as evidenced by subgroup analyses stratified by BMI. Moreover, combining exercise with dietary alterations yielded a significant reduction in VFA in male subjects, whereas no such effect was observed in female subjects. These 2 findings are critically interconnected, as they illuminate the differential responsiveness of individuals with obesity, irrespective of gender, to interventions that incorporate dietary adjustments and physical activity aimed at mitigating visceral fat accumulation. Given the inclusion of participants with or without chronic diseases, future studies should further explore the potential differential effects of exercise and dietary interventions on ectopic fat in these populations to enhance our understanding of how chronic disease status may influence intervention outcomes.

### Future research directions

Future research should aim to explore several critical areas to deepen our understanding of the interplay between exercise and diet on obesity-related outcomes. Specifically, studies should investigate the factors influencing the variability of physical exercise interventions, including differences in duration, intensity, and individual fitness levels, in conjunction with variations in dietary composition and types. Additionally, research into the underlying mechanisms driving the formation of ectopic fat, as well as the associated metabolic and hormonal changes, is necessary to identify potential therapeutic targets. Moreover, to identify the combined effects of the Mediterranean diet and exercise training on ectopic fat, it is essential to investigate the mechanism of the impact of the Mediterranean diet and its fatty acid composition on intramuscular triacylglycerols. Ethnicity is another important factor that warrants attention, as it may influence the response to combined exercise and dietary interventions. Furthermore, variability in the methodologies used across studies, such as differing measurement techniques for body composition and visceral fat, should be systematically analyzed to ensure consistency and comparability of findings. Addressing these research gaps will provide a more comprehensive understanding and guide the development of tailored interventions for diverse populations.

## Author contributions

The authors’ responsibilities were as follows – FK, MHM, MM: carried out the screenings and reviews; FK: carried out the analysis of the articles; FK, MN, SB, DMC, SM, RB: drafted and revised the manuscript; and all authors: read and approved the final manuscript.

## Data availability

The data that support the findings of this study are available from the corresponding author on request.

## Funding

The authors reported no funding received for this study.

## Conflict of interest

FK reports that administrative support was provided by the University of Kashan and relationship with the University of Kashan that includes employment. All other authors report no conflicts of interest.
