# Supplementary material for: The Impact of Exercise Training Plus Dietary Interventions on Ectopic Fat in Population with Overweight/Obesity with and without Chronic Disease: A Systematic Review, Meta-analysis, and Metaregression of Randomized Clinical Trials
Source: Curr Dev Nutr. 2025 Feb 21;9(4):104574. doi: 10.1016/j.cdnut.2025.104574 (PMC11964600; doi:10.1016/j.cdnut.2025.104574)
Supplement: multimedia component 1 [file mmc1.docx]

**The impact of exercise training plus dietary interventions on ectopic fat in overweight/obese population with and without chronic disease: a systematic review, meta‐analysis, and meta-regression of randomized clinical trials**

Fatemeh Kazeminasab

| **Supplementary Table 1.** Risk of bias assessment (PEDro scale) | | | | | | | | | | |
| --- | --- | --- | --- | --- | --- | --- | --- | --- | --- | --- |
| **Authors and Year of Publication** | **Criteria 1** | **Criteria 2** | **Criteria 3** | **Criteria 4** | **Criteria 5** | **Criteria 6** | **Criteria 7** | **Criteria 8** | **Criteria 9** | **Total** |
| Brennan et al. (2022) (51) | 🗸 | 🗸 | 🗴 | 🗸 | 🗸 | 🗸 | 🗸 | 🗸 | 🗸 | **8** |
| Brinkley et al. (2011) (68) | 🗸 | 🗸 | 🗴 | 🗸 | 🗴 | 🗸 | 🗸 | 🗸 | 🗸 | **7** |
| Cheng et al. (2017) (52) | 🗸 | 🗸 | 🗴 | 🗸 | 🗸 | 🗸 | 🗸 | 🗸 | 🗸 | **8** |
| Chorell et al. (2021) (53) | 🗸 | 🗸 | 🗴 | 🗸 | 🗴 | 🗸 | 🗸 | 🗸 | 🗸 | **7** |
| Cooper et al. (2012) (54) | 🗸 | 🗸 | 🗴 | 🗸 | 🗸 | 🗸 | 🗸 | 🗸 | 🗸 | **8** |
| Ezpeleta et al. (2023) (55) | 🗸 | 🗸 | 🗴 | 🗸 | 🗸 | 🗸 | 🗴 | 🗸 | 🗸 | **7** |
| Fayh et al. (2013) (56) | 🗸 | 🗸 | 🗴 | 🗸 | 🗸 | 🗸 | 🗸 | 🗸 | 🗸 | **8** |
| Ge et al. (2014) (50) | 🗸 | 🗸 | 🗴 | 🗸 | 🗸 | 🗸 | 🗸 | 🗸 | 🗸 | **8** |
| Gepner et al. (2018) (48) | 🗸 | 🗸 | 🗴 | 🗸 | 🗸 | 🗴 | 🗸 | 🗸 | 🗸 | **7** |
| Ghitea et al. (2021) (57) | 🗸 | 🗸 | 🗴 | 🗸 | 🗴 | 🗴 | 🗸 | 🗸 | 🗸 | **6** |
| Goodpaster et al. (2010) (58) | 🗸 | 🗸 | 🗴 | 🗸 | 🗸 | 🗴 | 🗸 | 🗸 | 🗸 | **7** |
| Hays et al. (2006) (59) | 🗸 | 🗸 | 🗴 | 🗸 | 🗴 | 🗸 | 🗴 | 🗸 | 🗸 | **6** |
| Hens et al. (2021) (60) | 🗸 | 🗸 | 🗸 | 🗸 | 🗸 | 🗴 | 🗸 | 🗸 | 🗸 | 8 |
| Idoate et al. (2010) (69) | 🗸 | 🗸 | 🗴 | 🗴 | 🗸 | 🗸 | 🗸 | 🗸 | 🗸 | **7** |
| Janssen et al. (2002) (70) | 🗸 | 🗸 | 🗴 | 🗸 | 🗸 | 🗸 | 🗸 | 🗸 | 🗸 | **8** |
| Larson et al. (2010) (61) | 🗸 | 🗸 | 🗴 | 🗸 | 🗸 | 🗸 | 🗸 | 🗸 | 🗸 | **8** |
| Larson-Meyer et al. (2006) (49) | 🗸 | 🗸 | 🗴 | 🗸 | 🗸 | 🗸 | 🗸 | 🗸 | 🗸 | **8** |
| Abbate et al. (2021) (71) | 🗸 | 🗸 | 🗴 | 🗸 | 🗸 | 🗸 | 🗸 | 🗸 | 🗸 | **8** |
| Nicklas et al. (2009) (72) | 🗸 | 🗸 | 🗴 | 🗸 | 🗸 | 🗸 | 🗸 | 🗸 | 🗸 | **8** |
| Oh et al. (2014) (76) | 🗸 | 🗸 | 🗸 | 🗸 | 🗸 | 🗸 | 🗸 | 🗸 | 🗸 | **9** |
| Okura et al (2007) (73) | 🗸 | 🗸 | 🗴 | 🗸 | 🗸 | 🗸 | 🗸 | 🗸 | 🗸 | **8** |
| Otten et al. (2018) (62) | 🗸 | 🗸 | 🗸 | 🗸 | 🗸 | 🗸 | 🗸 | 🗸 | 🗸 | **9** |
| Otten et al. (2019) (74) | 🗸 | 🗸 | 🗴 | 🗸 | 🗸 | 🗸 | 🗸 | 🗸 | 🗸 | **8** |
| Redman et al. (2010) (65) | 🗸 | 🗸 | 🗴 | 🗸 | 🗴 | 🗴 | 🗸 | 🗸 | 🗸 | **6** |
| Rice et al. (1999) (77) | 🗸 | 🗸 | 🗴 | 🗸 | 🗸 | 🗸 | 🗸 | 🗸 | 🗸 | **8** |
| Ross et al. (1996) (78) | 🗸 | 🗸 | 🗴 | 🗸 | 🗴 | 🗸 | 🗸 | 🗸 | 🗸 | **7** |
| Shah et al. (2009) (63) | 🗸 | 🗸 | 🗴 | 🗸 | 🗸 | 🗸 | 🗸 | 🗸 | 🗸 | **8** |
| Snel et al. (2012) (64) | 🗸 | 🗸 | 🗴 | 🗸 | 🗴 | 🗸 | 🗸 | 🗸 | 🗸 | **7** |
| Tamura et al. (2005) (47) | 🗸 | 🗸 | 🗴 | 🗸 | 🗸 | 🗸 | 🗸 | 🗸 | 🗸 | **8** |
| Toledo et al. (2008) (66) | 🗸 | 🗸 | 🗴 | 🗸 | 🗸 | 🗸 | 🗸 | 🗸 | 🗸 | **8** |
| Yoshimura et al. (2014) (67) | 🗸 | 🗸 | 🗴 | 🗴 | 🗸 | 🗸 | 🗸 | 🗸 | 🗸 | **7** |
| You et al. (2004) (75) | 🗸 | 🗸 | 🗴 | 🗸 | 🗴 | 🗸 | 🗸 | 🗸 | 🗸 | **7** |


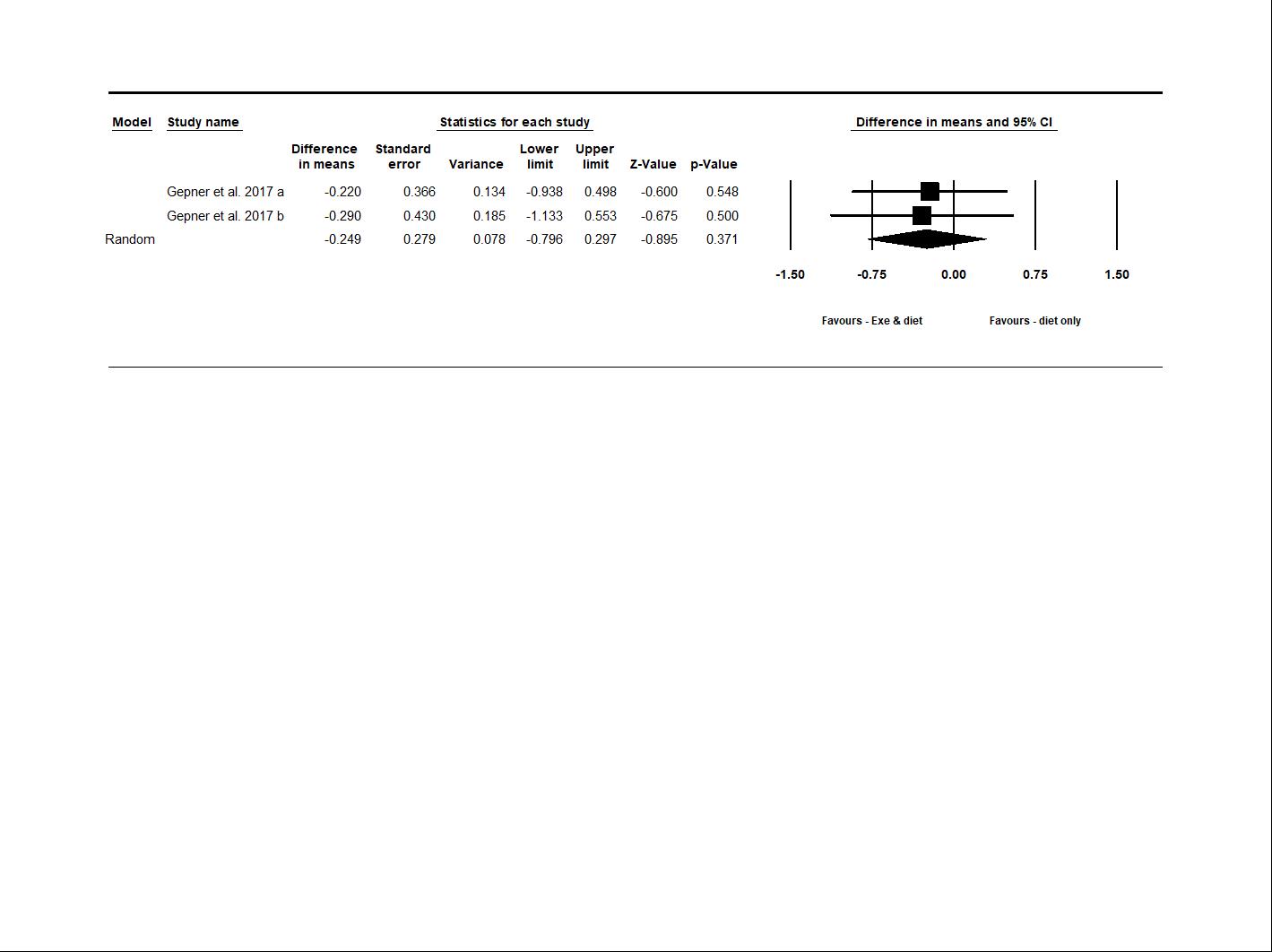


Supplementary Figure 1. Forest plot of the effects of exercise and diet vs. diet only on pancreatic fat. Data are reported as WMD (95% confidence limits). WMD, weighted mean difference


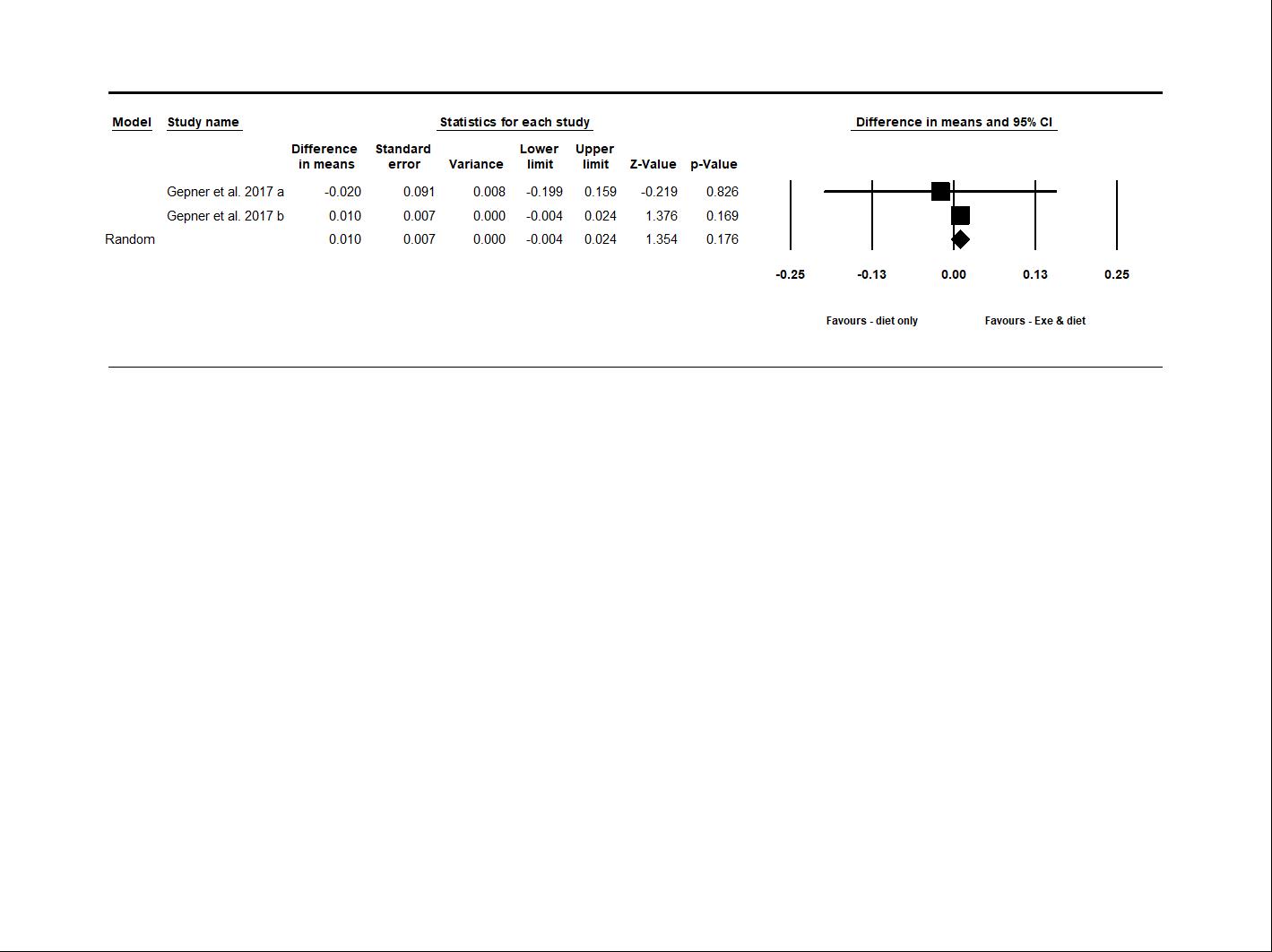


Supplementary Figure 2. Forest plot of the effects of exercise and diet vs. diet only on renal sinus fat. Data are reported as WMD (95% confidence limits). WMD, weighted mean difference
